# Engineering a vector-based pan-*Leishmania* vaccine for humans: proof of principle

**DOI:** 10.1038/s41598-020-75410-0

**Published:** 2020-10-29

**Authors:** Pedro Cecílio, James Oristian, Claudio Meneses, Tiago D. Serafim, Jesus G. Valenzuela, Anabela Cordeiro da Silva, Fabiano Oliveira

**Affiliations:** 1 i3S - Instituto de Investigação e Inovação em Saúde, Universidade do Porto, Porto, Portugal; 2 Parasite Disease Group, IBMC - Instituto de Biologia Molecular e Celular, Universidade do Porto, Rua Alfredo Allen, 208, 4200-135 Porto, Portugal; 3 Departamento de Ciências Biológicas, Faculdade de Farmácia da Universidade do Porto (FFUP), Porto, Portugal; 4 Vector Molecular Biology Section, Laboratory of Malaria and Vector Research, National Institute of Allergy and Infectious Diseases, National Institutes of Health, 12735 Twinbrook Parkway, Rockville, MD 20852 USA

**Keywords:** Vaccines, Parasitic infection, Parasitology

## Abstract

Leishmaniasis is a spectrum of diseases transmitted by sand fly vectors that deposit *Leishmania* spp. parasites in the host skin during blood feeding. Currently, available treatment options are limited, associated with high toxicity and emerging resistance. Even though a vaccine for human leishmaniasis is considered an achievable goal, to date we still do not have one available, a consequence (amongst other factors) of a lack of pre-clinical to clinical translatability. Pre-exposure to uninfected sand fly bites or immunization with defined sand fly salivary proteins was shown to negatively impact infection. Still, cross-protection reports are rare and dependent on the phylogenetic proximity of the sand fly species, meaning that the applicability of a sand fly saliva-based vaccine will be limited to a defined geography, one parasite species and one form of leishmaniasis. As a proof of principle of a future vector saliva-based pan-*Leishmania* vaccine, we engineered through a reverse vaccinology approach that maximizes translation to humans, a fusion protein consisting of immunogenic portions of PdSP15 and LJL143, sand fly salivary proteins demonstrated as potential vaccine candidates against cutaneous and visceral leishmaniasis, respectively. The in silico analysis was validated ex vivo, through T cell proliferation experiments, proving that the fusion protein (administered as a DNA vaccine) maintained the immunogenicity of both PdSP15 and LJL143. Additionally, while no significant effect was detected in the context of *L. major* transmission by *P. duboscqi*, this DNA vaccine was defined as partially protective, in the context of *L. major* transmission by *L. longipalpis* sand flies. Importantly, a high IFNγ response alone was not enough to confer protection, that mainly correlated with low T cell mediated *Leishmania*-specific IL-4 and IL-10 responses, and consequently with high pro/anti-inflammatory cytokine ratios. Overall our immunogenicity data suggests that to design a potentially safe vector-based pan-*Leishmania* vaccine, without geographic restrictions and against all forms of leishmaniasis is an achievable goal. This is why we propose our approach as a proof-of principle, perhaps not only applicable to the anti-*Leishmania* vector-based vaccines’ field, but also to other branches of knowledge that require the design of multi-epitope T cell vaccines with a higher potential for translation.

## Introduction

Leishmaniasis is a spectrum of diseases caused by at least 20 different species belonging to the *Leishmania* genus (Kinetoplastida; Trypanosomatida)^1^. These parasites have a complex life-cycle that depends on two different organisms to be completed: an arthropod vector (phlebotomine sand flies) and a vertebrate host (from reptiles to mammals)^1^. Furthermore, considering the parasite-host binomium: most human *Leishmania* pathogens also infect animals (zoonosis)^1,2^; disease development is dependent on the evasion/manipulation of the host immune response^3^; and the disease forms usually correlate with specific parasite species^1^. Additionally, considering the parasite-vector interface, although experimentally some vectors were shown to be permissive to different *Leishmania* spp., in nature ecological and molecular factors typically restrict vector-*Leishmania* spp. pairings^1,4,5^. All of these considerations make the control of leishmaniasis an extremely challenging task, requiring a trinomial focus: on the infected human individuals; on the vectors; and on the different animal reservoirs^6,7^.

On opposite sides of the leishmaniasis disease spectrum are the cutaneous and visceral forms, both very relevant, due to the number of cases recorded worldwide annually (and DALYs associated), considering the former^8,9^, or due to the fatality rate (over 95%, if untreated), considering the latter^9^. Together, visceral and cutaneous leishmaniases are endemic in 97 countries (co-endemic in 65 of them) and are responsible for up to one million new cases and more than 20,000 deaths, estimated to occur annually^9,10^. Although visceral leishmaniasis was defined as potentially controllable in the short-term, today this is only hypothetically achievable in the Indian sub-continent where the disease is of anthroponotic origin^7,11^. Despite several attempts to prevent the disease, with numerous candidates tested along the years, today there is still no vaccine available for human leishmaniasis^12^. The absence of prophylaxis makes disease control dependent on the treatment of active cases. However, not only is a vaccine for leishmaniasis considered an achievable goal, justified by the protection observed following the leishmanization campaigns^12^, but also an essential tool to meet the ultimate target of disease control and even elimination, considering a long-term perspective^7^.

One of the most recently explored possibilities of “anti-*Leishmania*” vaccines are the vector-based ones. An anti-vector-saliva immune response, elicited by exposure to uninfected sand fly bites, conferred protection against vector-transmitted cutaneous disease^13^, opening the door to testing defined sand fly-salivary proteins as vaccines that demonstrated effectiveness in the context of cutaneous^14–18^ or visceral^19^ disease. Protection is thought to be mediated by saliva-specific IFN-γ secretion by CD4 + T cells at the bite site, early after transmission, via the generation of a robust Th1 delayed-type hypersensitivity (DTH) reaction, that negatively (and indirectly) affects parasites’ establishment^20^. Still, cross-protection reports are rare and dependent on phylogenetic proximity of the sand fly species^21^ (less probable to occur in New World *versus* Old World vector species), meaning that most of the sand fly salivary proteins will probably work as effective vaccines only in a defined geography, against one parasite species and consequently one form of leishmaniasis^22^. Additionally, most of these proteins have potent biological activities^23^, which may pose a barrier considering the safety requirements for approval of a human pharmaceutical^24^.

Having in mind the above-mentioned limitations, we engineered a chimeric antigen that includes in silico-determined immunogenic T-cell epitopes from two salivary proteins whose anti-*Leishmania* vaccine potential was at least partially demonstrated: PdSP15 from *Phlebotomus duboscqi*, the main West African vector of cutaneous leishmaniasis, a protein defined as an effective anti-*Leishmania* vaccine in a non-human primate model^18^; and LJL-143 from *Lutzomyia longipalpis*, the main South American vector of visceral leishmaniasis, an antigen demonstrated to negatively impact *L. infantum* parasites ex vivo^25^, and to potentially work as “adjuvant-like” in a prime-boost immunization strategy^26^. We further validated the overall bioinformatics predictions ex vivo and show that this neo-antigen, administered as a DNA vaccine, generates specific T cell reactivity against salivary gland extracts from both *P. duboscqi* and *L. longipalpis*, indicating a potential broadening of the spectrum. Finally, we tested this new potential vaccine in the context of *L. major* transmission by *P. duboscqi* or *L. longipalpis*, and propose our strategy as a proof of principle for a future vector-derived pan-*Leishmania* vaccine: a single antigen, constructed modularly with fractions of distinct antigens, and rationally using reverse vaccinology.

## Results

### Epitope mapping of the sand fly salivary proteins

Going towards a reverse vaccinology approach we predicted CD4 + and CD8 + T cell epitopes considering both murine and human alleles for two distinct sand fly salivary proteins previously studied in the context of anti-*Leishmania* vaccines, PdSP15 from *P. duboscqi* and LJL-143 from *L. longipalpis*, in a way to disclose their immunogenic potential considering cell-mediated responses. We performed this mouse/human analysis to bridge pre-clinical and clinical contexts. Additionally, we also mapped CD4 + T cell epitopes, considering only human alleles, for two known homologs of the aforementioned proteins, PpeSP06 and PpSP15 respectively, to assess the potential for cross-reactivity.

**Figure 1:**
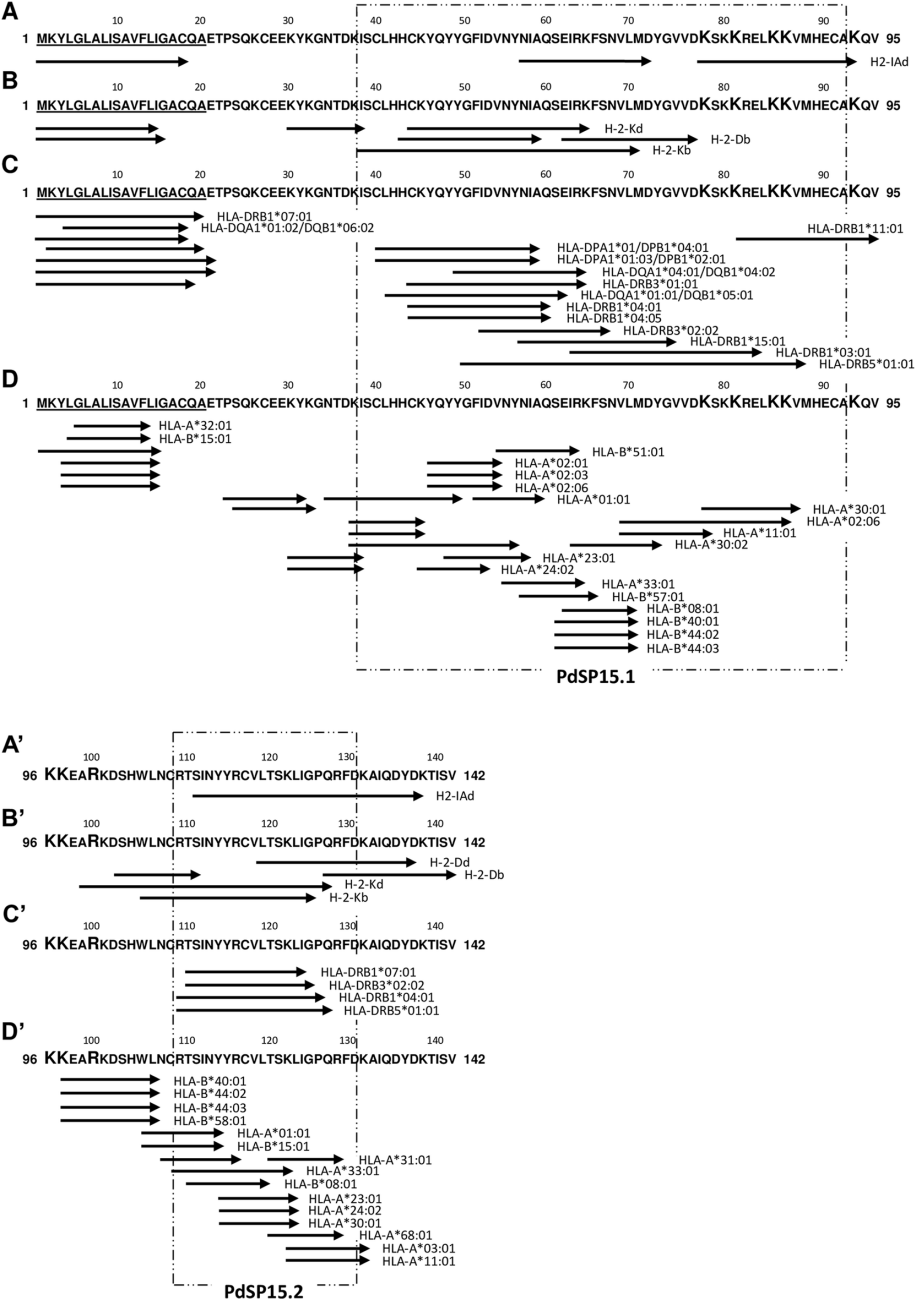
PdSP15 T cell epitope mapping: overall picture of the in silico analysis performed against murine and human MHC-I and MHC-II molecules. PdSP15 (GenBank Acc. No. ABI15933) T cell epitope mapping was performed using the IEDB Analysis Resource considering 3 murine and 27 human MHC-II alleles and 5 murine and 27 human MHC-I alleles. The translated results of the predictions of murine MHC-II restricted epitopes (**A**,**A′**), murine MHC-I restricted epitopes (**B**,**B′**), human MHC-II restricted epitopes (**C**,**C′**) and human MHC-I restricted epitopes (**D**,**D′**) are represented. Results are shown by allele. Each arrow represents one or more (contiguous) predicted epitopes: the top 1% hits for MHC-I molecules and the top 2.5% (very high affinity binders) or 10% hits for MHC-II human and murine alleles, respectively. The underlined protein residues represent the signal peptide sequence. The magnified protein residues are potentially important for protein biological activity. Dashed boxes represent the convergent analysis of the different in silico determinations that enabled the selection of two protein portions to be included in the final chimeric sand fly salivary antigen.

Figure 1 illustrates the overall snapshot obtained for PdSP15, where there are highlighted the protein regions containing epitopes predicted to bind to MHC-II murine alleles (A and A′), MHC-I murine alleles (B and B′), MHC-II human alleles (C and C′—only very high affinity binders, with a percentile rank ≤ 2.5, are represented) and MHC-I human alleles (D and D′). With respect to the MHC-II binding analysis against murine alleles, the predictions were negative for two of the three MHC molecules studied: H2-IE^d^, representative of BALB/c mice with the lowest percentile rank determined of 17.39 (Supplementary Data 1), and H2-IA^b^ representative of C57BL/6 mice with the lowest percentile rank determined of 18.55 (Supplementary Data 2). Only for the allele H2-IA^d^ from BALB/c, 14 epitopes were predicted as good binders (percentile rank ≤ 10; Supplementary Data 1), 4 of which within the signal peptide sequence, and the remaining in three different regions of the protein, represented in Fig. 1A/A′. Regarding binding predictions against MHC-I murine alleles, several 8–14 MER peptides were determined to bind efficiently (percentile rank ≤ 1) to four of the five alleles explored, two from BALB/c (H-2-D^d^ and H-2-K^d^, but not H-2-L^d^; Supplementary Data 3) and the other two from C57BL/6 (H-2-D^b^ and H-2-K^b^; Supplementary Data 4) mice, mainly within two regions we define as murine CD8 + “T cell epitopes’ rich”: between residues 40 and 80 (Fig. 1B), and the C-terminal portion, starting from residue 100 (Fig. 1B′). The analysis against MHC-II human alleles also revealed, apart from several hits in the signal peptide region, two “T cell epitopes’ rich” protein regions (Fig. 1C/C′). Considering only the determined very high affinity binders (percentile rank ≤ 2.5), peptides comprised between residues 40 and 80 were predicted to bind against 11 different human alleles, while peptides comprised between residues 110 and 130 were predicted to bind against 4 different human alleles (Fig. 1C/C′; Supplementary Data 5, represented in dark green). If we extend this analysis to the peptides with determined percentile ranks between 2.5 and 7.5, these two “highly immunogenic” regions (as predicted in silico considering cellular immunity) are still noticeable (Supplementary Figure 2A/A′; Supplementary Data 5, represented in light green). Considering this analysis, the same two regions now contain peptides predicted to bind to 17 and 12 different human alleles, respectively (Supplementary Figure 2A/A′). Importantly, only for 3 of the 27 human MHC-II molecules studied, no CD4 + T cell epitopes were predicted, if we exclude the signal peptide sequence: HLA-DQA1*03:01/DQB1*03:02; HLA-DQA1*05:01/DQB1*03:01 and HLA-DRB1*12:01 (Supplementary Data 5). The same human MHC-II binding analysis was performed for the PdSP15 homologue, PpSP15, with a similar determined overall picture (Supplementary Figure 3A/A′; Supplementary Data 6). Briefly, excluding the signal peptide sequence, two similar “highly immunogenic” regions were detected: considering all hits with determined percentile rank ≤ 7.5, peptides comprised between residues 40 and 80 were predicted to bind against 22 different human alleles (Supplementary Figure 3A; Supplementary Data 6), while peptides comprised between residues 110 and 130 were predicted to bind against 6 different human alleles (Supplementary Figure 3A′; Supplementary Data 6). Finally, regarding PdSP15 epitope mapping considering human MHC-I alleles, the analysis revealed many different epitopes, covering almost the entire protein sequence. Still, most of these epitopes were localized within the same above-mentioned two regions, with combined determined good binders (percentile rank ≤ 1) against 20 different human alleles (Fig. 1D/D′; Supplementary Data 7). From the remaining 7 alleles, no predicted binders were found for HLA-A*26:01, HLA-A*68:02, HLA-B*07:02, HLA-B*35:01 and HLA-B*53:01 (Supplementary Data 7).

**Figure 2:**
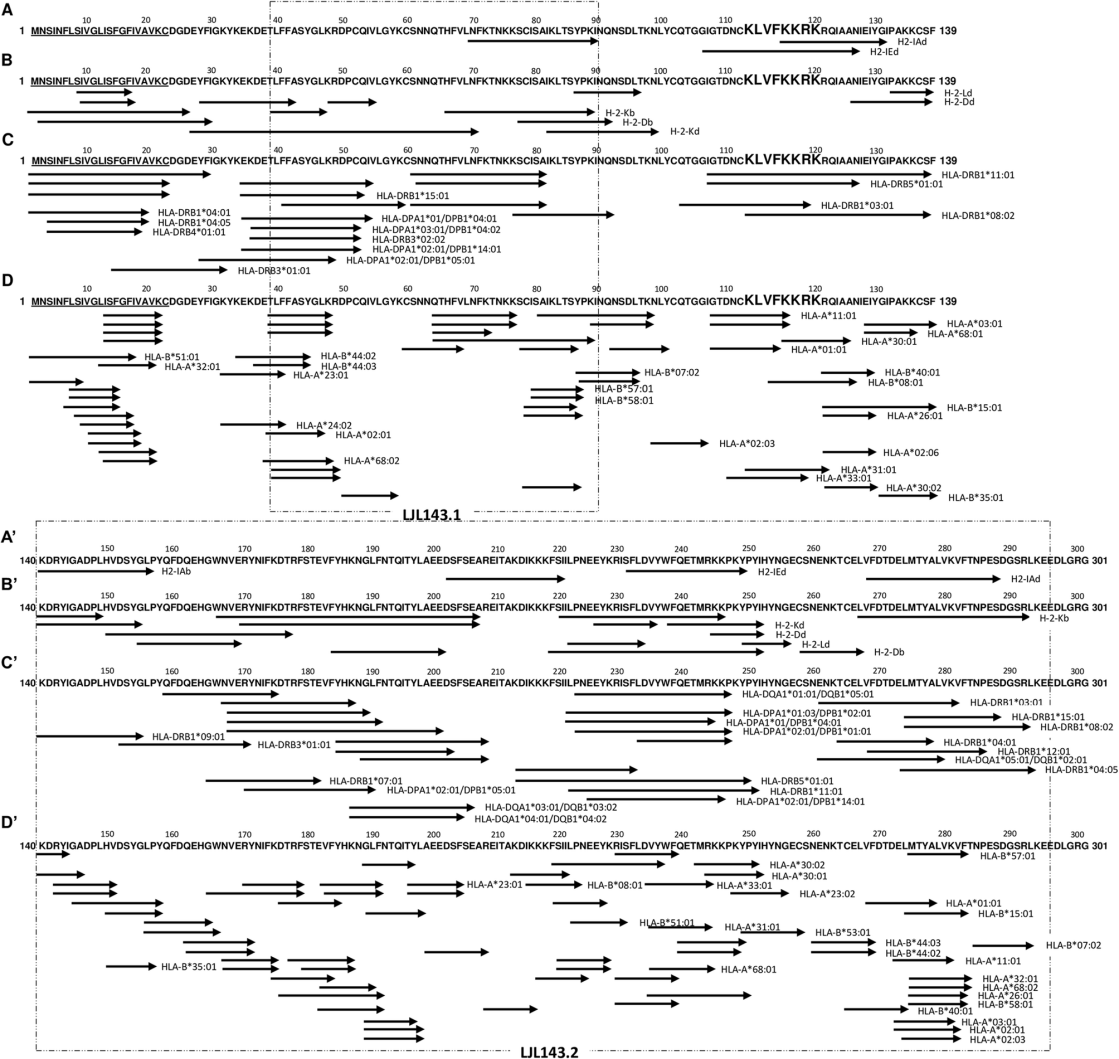
LJL-143T cell epitope mapping: overall picture of the in silico analysis performed against murine and human MHC-I and MHC-II molecules. LJL-143 (GenBank acc. No. AAS05319) T cell epitope mapping was performed using the IEDB Analysis Resource considering 3 murine and 27 human MHC-II alleles and 5 murine and 27 human MHC-I alleles. The translated results of the predictions of murine MHC-II restricted epitopes (**A,A′**), murine MHC-I restricted epitopes (**B**,**B′**), human MHC-II restricted epitopes (**C**,**C′**) and human MHC-I restricted epitopes (**D**,**D′**) are represented. Results are presented by allele. Each arrow represents one or more (contiguous) predicted epitopes: the top 1% hits for MHC-I molecules and the top 2.5% (very high affinity binders) or 10% hits for MHC-II human and murine alleles, respectively. The underlined protein residues represent the signal peptide sequence. The magnified protein residues are potentially important for protein biological activity. Dashed boxes represent the convergent analysis of the different in silico determinations that enabled the selection of two protein portions to be included in the final chimeric sandfly salivary antigen.

**Figure 3:**
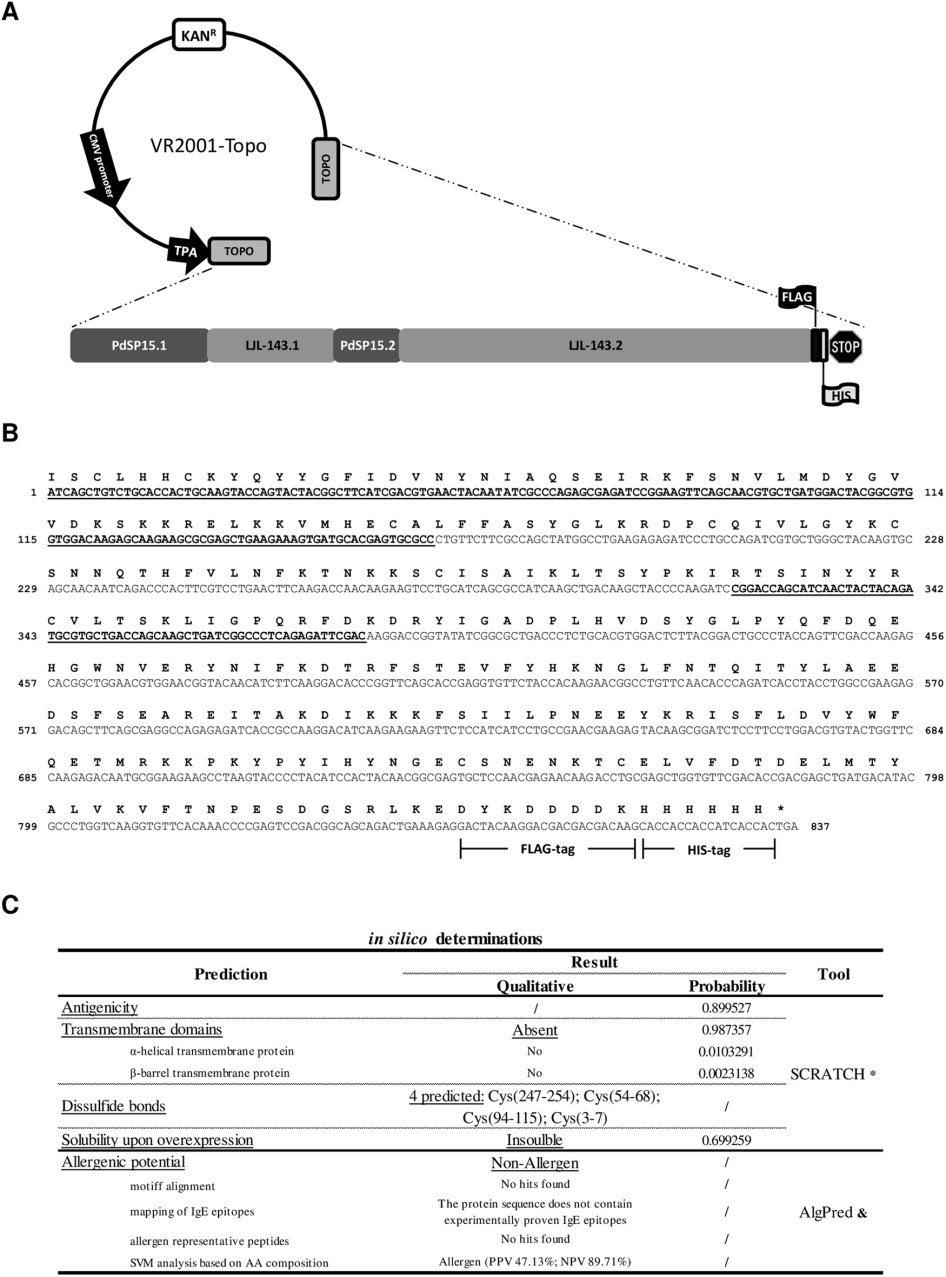
Engineering a multi-sand fly species based anti-*Leishmania* vaccine. A single DNA-based vaccine was engineered, considering a reverse vaccinology approach applied to the two salivary proteins this study focuses on: PdSP15 and LJL-143. The scheme of the final DNA vaccine, consisting of the VR2001-TOPO vector backbone and the fusion salivary antigen coding sequence, composed by the selected immunogenic portions of the two salivary proteins joined contiguously in an interpolated manner, a FLAG-tag, a HIS-tag and a stop codon is represented (**A**). The final sequence coding for the fusion salivary antigen (codon optimized for the mammalian codon usage bias), used in the cloning approach, as well as the respective translation, is shown (**B**). The underlined nucleotides code for the portions of PdSP15, while the non-underlined ones code for the portions of LJL-143. FLAG- and HIS-tags coding sequences are highlighted. The fusion protein amino-acid sequence was subjected to in silico predictions of antigenicity, solubility upon overexpression, presence of transmembrane domains, disulphide bonds formation and allergenic potential, whose quantitative and/or qualitative results are represented (**C**).

Similarly, Fig. 2 illustrates the results obtained for LJL-143. In the binding analysis against murine MHC-II alleles, peptides were predicted to bind against the two BALB/c mice alleles screened (Supplementary Data 8) and against the C57BL/6 mice allele studied (Supplementary Data 9). In theory, the chances of immunogenicity (considering CD4 + T cell responses) will be higher in the BALB/c background, with five immunogenic regions determined, comparing with the C57BL/6 background, with only one region determined between residues 139 and 156 (Fig. 2A/A′; Supplementary Datas 8 and 9). Regarding the murine MHC-I restricted epitope mapping, several hits were determined to bind against the five alleles studied (Supplementary Datas 10 and 11 represent the results obtained for BALB/c and C57BL/6 alleles, respectively). Considering the five alleles together, the protein coverage in terms of predicted epitopes is almost integral, excluding the region between residues 100 and 130 (Fig. 2B/B′). With respect to the in silico analysis for human MHC-II alleles, and considering only the very high affinity binders (percentile rank ≤ 2.5), at least one epitope was predicted for 23 of the 27 alleles studied (Supplementary Data 12, dark green highlighted cells). This analysis showed once more an almost complete protein coverage of human MHC-II restricted epitopes (Fig. 2C/C′). Nevertheless, the region comprised between residues 60 and 160 seems to be less enriched in human CD4 + T cell epitopes with very high affinity binders determined only for 5/27 alleles (Fig. 2C/C′). If we extend the analysis and consider the peptides with determined percentile ranks between 2.5 and 7.5 (Supplementary Data 12, light green highlighted cells), the in silico predictions suggest that LJL-143 is pan-immunogenic, with at least one epitope predicted for the each of the 27 human alleles studied (that theoretically ensure ˃ 99% of human population coverage). This extended analysis, translated into Supplementary Figure 4A/A′ shows a complete protein coverage of human CD4 + T cell epitopes, with the maintenance of the region “less enriched”, now smaller and comprised between residues 90 and 140. The same human MHC-II binding analysis was performed for the LJL-143 homologue, PpeSP06 (Supplementary Data 13; Supplementary Figure 5A/A′). Similar to what we observed for LJL-143 almost the full protein sequence contains human MHC-II restricted epitopes, considering a percentile rank threshold of 7.5 (Supplementary Figure 5A/A′). Additionally, and also in line with the observed for the PpeSP06 homologue, more hits were found in the signal peptide and c-terminal regions, while less hits were found in the region comprised between residues 90 and 140 (Supplementary Figure 5A/A′). Overall, the analysis performed shows that PpeSP06 contains human MHC-II restricted epitopes for 25 of the 27 molecules studied, excluding the signal peptide sequence (no hits found for the alleles HLA-DQA1*05:01/DQB1*03:01 and HLA-DQA1*01:02/DQB1*06:02; Supplementary Data 13). Finally, regarding LJL-143 epitope mapping considering human MHC-I alleles, the analysis shows once more an almost complete coverage of the protein sequence, this time homogeneously (no “rich or poor epitope regions”; Fig. 2D/D′). Interestingly, and as was observed in the analysis against human MHC-II alleles, at least one peptide was defined as a good binder for all of the 27 alleles studied (Supplementary Data 14).

**Figure 4:**
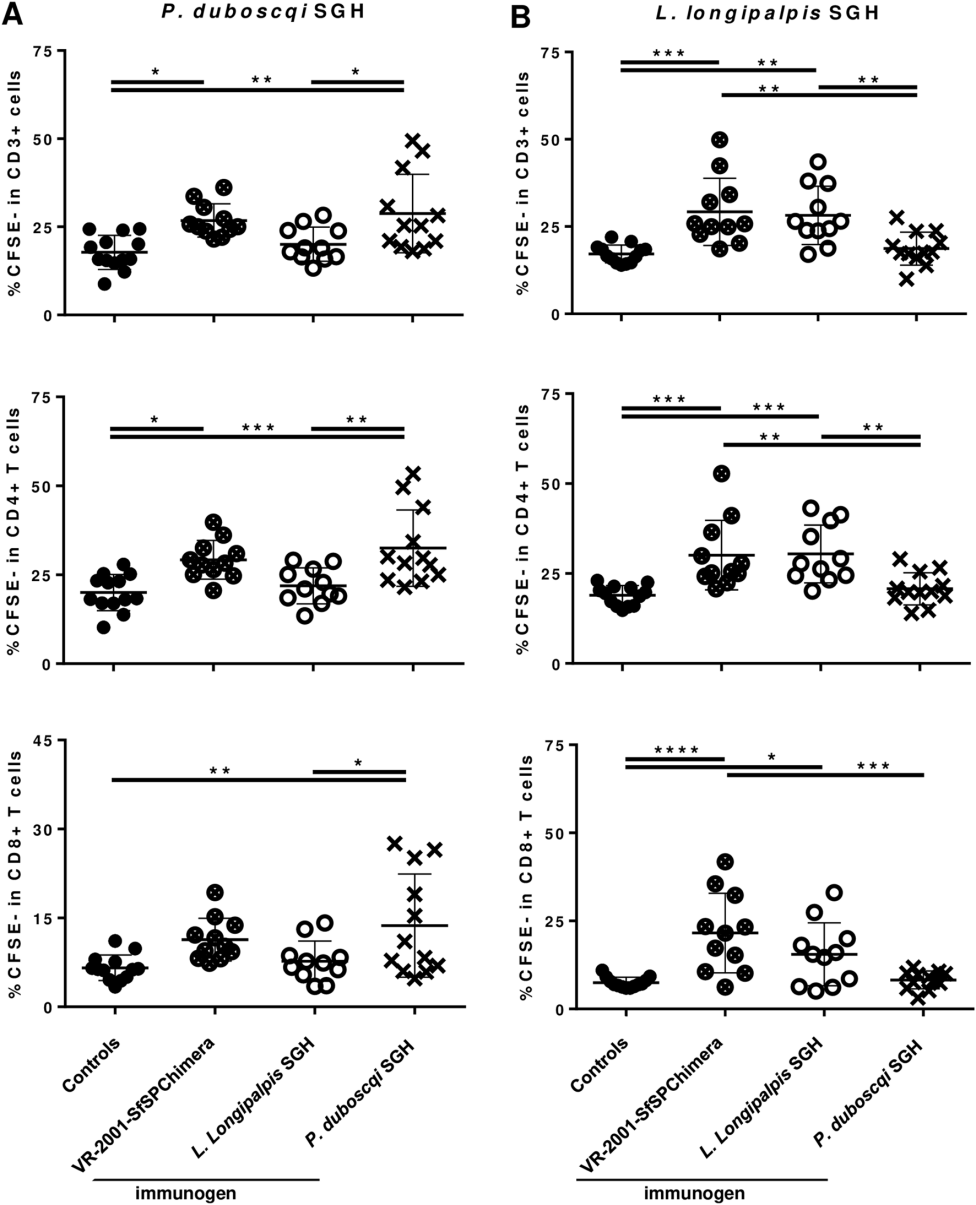
Vaccination with the sand fly-derived DNA chimeric vaccine elicits specific T cell responses against both *P. duboscqi* and *L. longipalpis* saliva. BALB/C mice were immunized intradermally in the right ear three times at 2 weeks intervals with 5 µg of VR-2001-SfSPChimera plasmid (crossed circles), or with either *L. longipalpis* (white circles) or *P. duboscqi* (crosses) SGH—the equivalent of 1 sand fly salivary gland pairs. Control animals received the same volume of the vehicle solution (PBS; black circles). One month after the last immunization, animals were euthanized, their spleens collected and processed to obtain CFSE-stained splenocytes suspensions. Frequencies of proliferating splenic T cells (total CD3 + and CD3 + /CD4 + or CD3 + /CD8 +) were determined by flow cytometry after 4 days of culture in the presence of BMDCs (5:1 ratio) pulsed with *P. duboscqi* (**A**) or *L. longipalpis* (**B**) SGH (final concentration of 3 sand fly salivary gland pair/ml). Results from three independent experiments are shown. Each dot represents one animal. Average and SD of the values within each group are shown. Statistical differences are properly identified (One-Way ANOVA with Tukey’s post hoc analysis: *p ≤ 0.05; **p ≤ 0.01; ***p ≤ 0.001 and ****p ≤ 0.0001).

**Figure 5:**
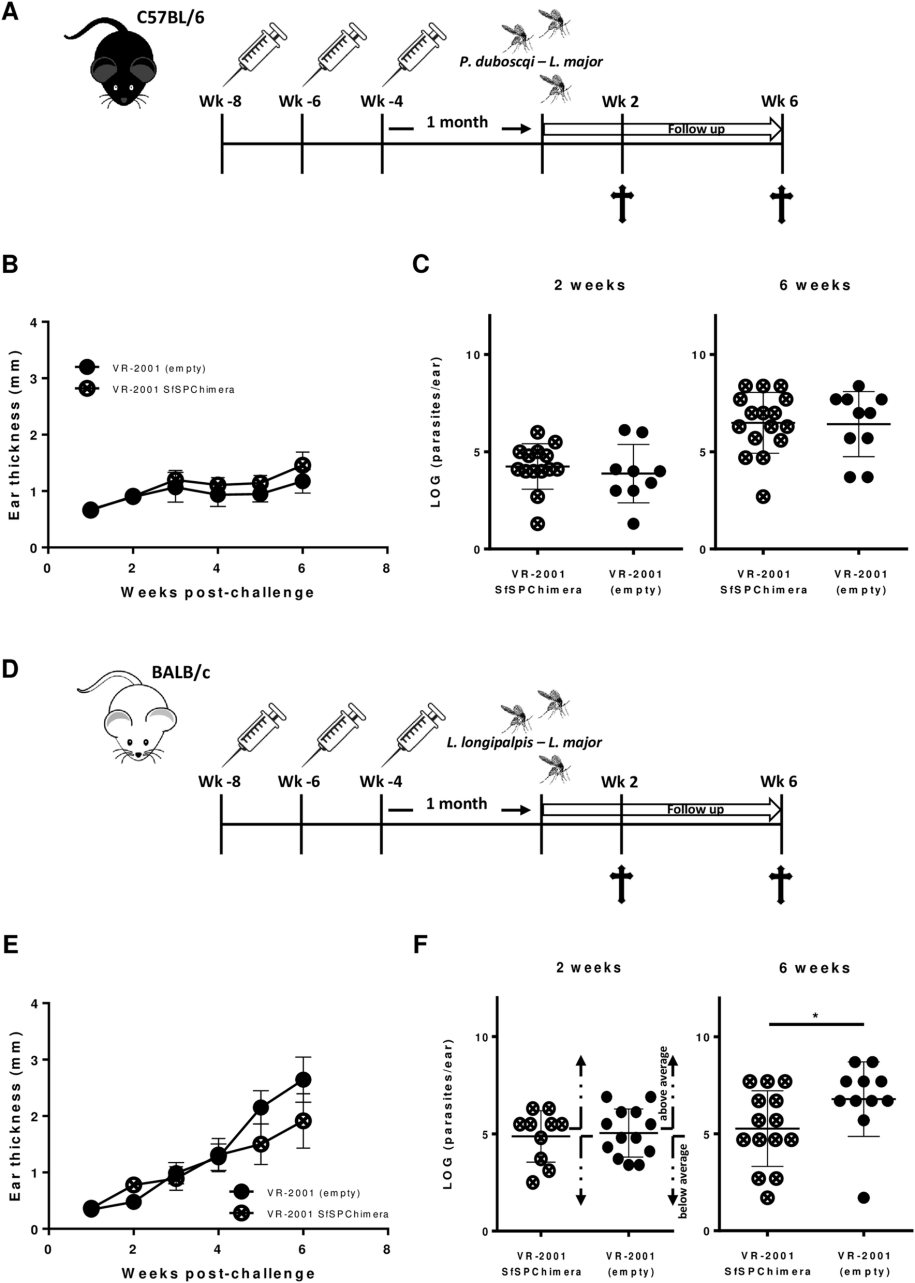
Sand fly-derived DNA chimeric vaccine preliminary efficacy trial. C57BL/6 or BALB/C mice were immunized intradermally in the right ear three times at 2 weeks intervals with 5 µg of VR-2001-SfSPChimera plasmid (crossed circles), or with the empty vector (control animals; black circles). C57BL/6 mice were challenged 1 month later on the contralateral ear via sand fly bites—10 *P. duboscqi* sand flies carrying mature *L. major* infections were used per animal. Ear pathology was assessed weekly, and experiments were terminated at either 2 or 6 weeks post challenge for parasite burden evaluation (**A**). Ear thickness measurements are represented for both VR-2001-SfSPChimera immunized and control animals (**B**). Total ear parasite burdens at 2 and 6 weeks post-challenge are also shown (**C**). BALB/c mice were also challenged 1 month after the last immunization on the contralateral ear via sand fly bites, using a different species—10 *L. longipalpis* sand flies carrying mature *L. major* infections were used per animal (**D**). The follow up and endpoints used were the same as above-mentioned (**D**). Ear thickness measurements are once more represented for both VR-2001-SfSPChimera immunized and control animals (**E**), as are the determined parasite burdens at 2 and 6 weeks post-challenge (**F**). Results from at least two independent experiments are represented in each graph. Each dot represents one animal. Average and SEM or SD of the values within each group are shown for ear thickness or parasite burden measurements, respectively. Each group represented in the 2 weeks’ time-point of panel F was subdivided in two groups based on parasite burden values: below and above average. Statistical differences are properly identified (Mann–Whitney test: *p ≤ 0.05).

In summary, in silico predictions indicate that both proteins have a good potential for immunogenicity, particularly considering human alleles.

### Engineering a multi-sand fly species based anti-Leishmania vaccine: proof of principle

Focusing on the in silico determinations described above we performed a convergent analysis to define the protein regions of both PdSP15 and LJL-143 with the “best immunogenic potential”: (1) containing both MHC-I and MHC-II restricted epitopes for different human alleles (to maximize population coverage); (2) containing conserved epitopes (comparing protein homologs, to maximize potential cross-protective potential); and (3) containing both murine MHC-I and MHC-II restricted epitopes (to maximize the possibility of translation of pre-clinical studies to the clinical context). Regarding PdSP15, this convergent analysis revealed two protein portions, PdSP15.1 and PdSP15.2, consisting of 55 and 22 amino acids, respectively (Fig. 1; dashed boxes). Although these portions comprise 54% or 63% excluding the signal peptide sequence, of the full-length protein, they overall contain the majority of the murine and human MHC-I and -II restricted epitopes predicted in silico (Fig. 1; Supplementary Figure 2). Additionally, they are 61.8% and 78.3% conserved, comparing with PpSP15, the PdSP15 homologue studied (Supplementary Figure 3), and exclude the signal peptide sequence, as well as 4 of the 8 basic residues thought to be important for substrate interaction (e.g. heparin), and consequently for the PdSP15 anti-haemostatic properties^27^. Similarly, for LJL-143 two protein portions were defined, LJL143.1 and LJL143.2, consisting of 50 and 155 amino acids, respectively (Fig. 2). Although visually less clear, the convergent analysis (dashed boxes) led us to exclude, in addition to the signal peptide, the region between residues 90 and 140, less enriched in human MHC-II restricted epitopes (Fig. 2; Supplementary Figure 4). LJL143.1 and LJL143.2 together represent 68% or 74% excluding the signal peptide sequence, of the full-length protein and are 38.5 and 50% conserved, comparing with PpeSP06, the LJL-143 homologue studied (Supplementary Figure 5).

These rationally chosen protein portions were then used as a template to design a single antigen (fusion protein) that in theory would maintain the individual immunogenic properties of each of the single sand fly salivary proteins, but not their biological activities. To maximize the potential loss of the known anti-haemostatic properties of PdSP15 and LJL-143^27,28^, we designed the fusion antigen in an interpolated manner (Fig. 3A). We did not use any linkers, and added a dual tag at the C-terminal (FLAG-tag followed by HIS-tag; Fig. 3A), to allow protein purification and traceability, as well as tags removal (the FLAG-tag contains an enterokinase cleavage site). The resulting sequence coding for this neo-antigen was codon optimized to the mammalian codon usage preference (Fig. 3B), synthetized and cloned in to the VR2001-TOPO vector, immediately downstream of a sequence coding for the tissue plasminogen activator signal peptide, that ensures protein secretion (Fig. 3A). The VR2001-TOPO vector was previously used both in the context of DNA vaccination and of in vitro protein expression using a mammalian expression system^25,29^. Furthermore, we submitted the fusion protein sequence to additional bioinformatics determinations using the available online tools SCRATCH and AlgPred. This engineered neo-antigen was defined as potentially immunogenic (with a probability of 89.9%, Fig. 3C). No transmembrane domains were predicted within the protein sequence, while 4 disulphide bonds were predicted to be formed, due to the presence of 8 cysteines (Fig. 3C). Additionally, the fusion protein was predicted to be insoluble under overexpression conditions, and a non-allergen, due to the 3 of 4 negative results given by the AlgPred algorithm (Fig. 3C).

In theory, the above-mentioned approach (we propose as a proof of principle of a future vector-derived pan anti-*Leishmania* vaccine) warrants the broadening of the vaccine spectrum of any individual sand fly salivary protein candidate, taking in consideration the geography (Old and New worlds), target population (endemics and non-endemics) and disease manifestation.

### Ex vivo cell proliferation experiments attest the immunogenicity of the engineered multi-sand fly species single-antigen and validate in silico determinations

To understand if the immunogenic properties of PdSP15 and LJL-143 were maintained in the context of the fusion antigen, we performed a DNA vaccination-based immunogenicity trial. Briefly, BALB/C mice, background we chosen based on in silico predictions, were immunized intradermally in the right ear three times at 2 weeks intervals with 5 µg of VR-2001-SfSPChimera plasmid, or with either *P. duboscqi* or *L. longipalpis* Salivary Gland Homogenates (SGH)—the equivalent of 1 sand fly salivary gland pair. Control animals received the same volume of the vehicle solution (Phosphate-Buffered Saline—PBS). One month after the last immunization, we evaluated T cell proliferation in the context of recall experiments using a co-culture of CFSE-stained splenocytes from immunized animals, and naïve BMDCs pulsed with *P. duboscqi*, *L. longipalpis*, *P. papatasi* or *P. pernicious* SGH at a final concentration of 3 sand fly salivary gland pairs/ml. Co-cultures of splenocytes and non-pulsed BMDCs, either non-stimulated or stimulated with the mitogen Concanavalin A, were used as experimental negative and positive controls, respectively. Importantly these controls revealed that the splenic T cells’ basal proliferation levels (Supplementary Figure 6A), and proliferative capacity (Supplementary Figure 6B) were comparable among the different groups. When we used *P. duboscqi* SGH as recall agent, we detected significantly higher specific T cell proliferation (CD3 +) in the groups immunized with either *P. duboscqi* SGH or the chimeric DNA vaccine, compared to control animals (Fig. 4A; *p* ≤ 0.01 and *p* ≤ 0.05, respectively), and to animals that received *L. longipalpis* SGH (Fig. 4A; *p* ≤ 0.05). This picture was overall reproduced when we analysed specific T helper (CD3 + /CD4 +) and T cytotoxic (CD3 + /CD8 +) cells’ proliferation separately, although more consistently for the first T cell subset. All statistically significant differences were maintained when we analysed proliferation within CD3 + /CD4 + cells (controls *versus P. duboscqi* SGH and VR-2001-SfSPChimera groups, *p* ≤ 0.001 and *p* ≤ 0.05, respectively; *P. duboscqi* SGH group *versus L. longipalpis* SGH group, *p* ≤ 0.01), while regarding CD3 + /CD8 + cells’ proliferation the statistical relevance comparing VR-2001-SfSPChimera and control groups was not observed (Fig. 4A). Importantly, when we used *L. longipalpis* SGH as a stimulus, we detected specific T cell proliferation (CD3 +) in the group of animals immunized with *L. longipalpis* SGH, and once again in animals that received the chimeric DNA vaccine (Fig. 4B). Relevantly, the proliferation levels were similar for these two groups, and significantly higher than both controls, and animals immunized with *P. duboscqi* SGH (Fig. 4B; at least *p* ≤ 0.01). Once more, this result was overall recapitulated when we analysed CD3 + /CD4 + and CD3 + /CD8 + populations individually (Fig. 4B). However CD3 + /CD8 + T cell proliferation in response to *L. longipalpis* was not significantly different comparing the groups that received *L. longipalpis* and *P. duboscqi* SGH (Fig. 4B). On the other hand, the responses detected against SGH from the vectors *P. papatasi* and *P. perniciosus*, that contain the homologous proteins PpSP15 and PpeSP06, respectively were not as striking. In response to *P. papatasi* SGH, a higher proliferation of CD4 + T cells was observed in the group of animals that was immunized with *P. duboscqi* SGH, in comparison to controls and to animals that received *L. longipalpis* SGH (Supplementary Figure 6C; *p* ≤ 0.05), but not compared to animals immunized with the chimeric DNA vaccine. In respect to the T cell proliferation detected in response to *P. perniciosus* SGH no major differences were observed (Supplementary Figure 6D).

**Figure 6:**
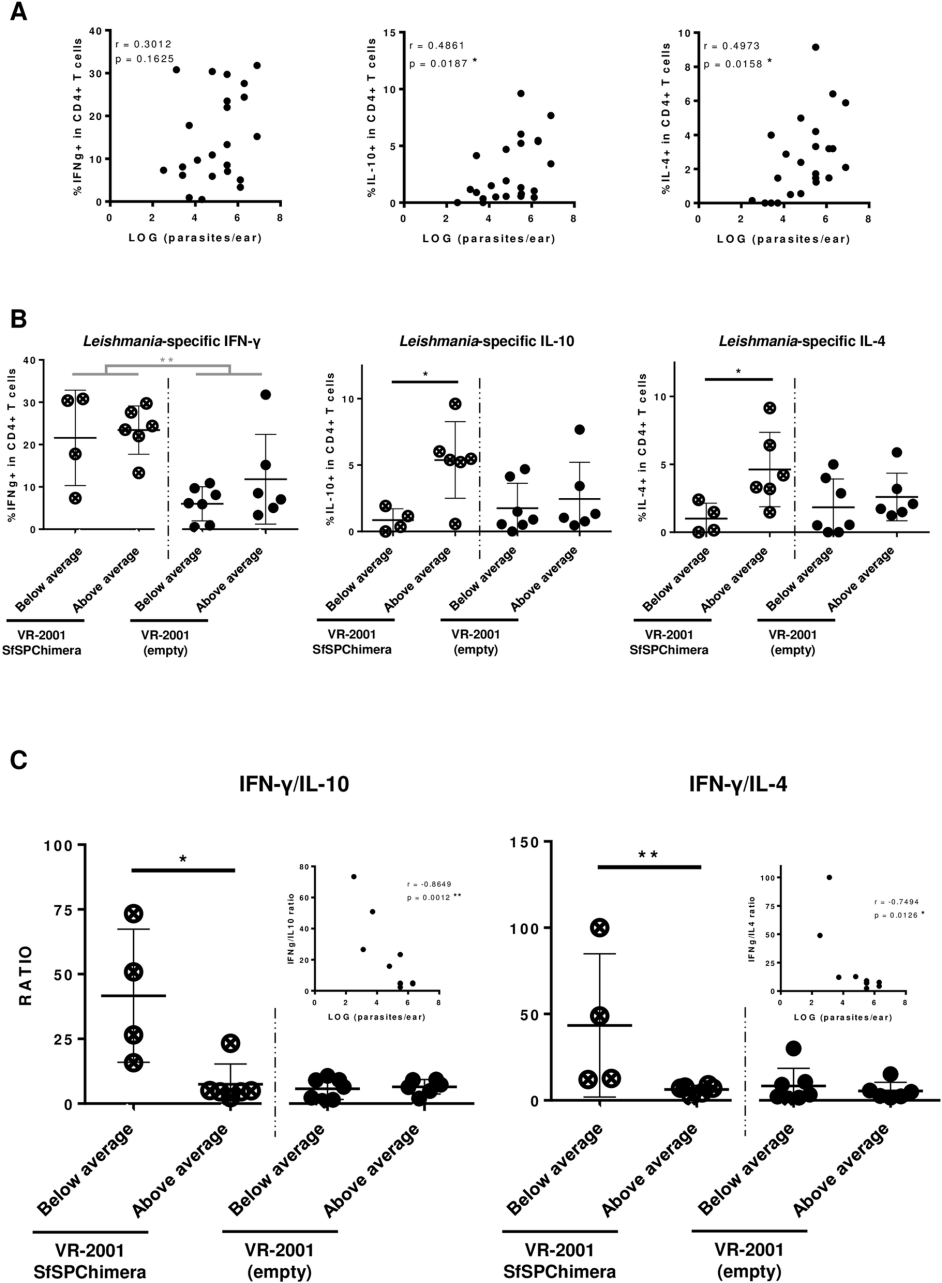
The protection against *L. longipalpis* sand fly-transmitted *L. major* infection in BALB/c mice is dependent on high IFN-γ/IL-10 and IFN-γ/IL-4 ratios. BALB/C mice were immunized intradermally in the right ear three times at 2 weeks intervals with 5 µg of VR-2001-SfSPChimera plasmid (crossed circles), or with the empty vector (control animals; black circles). One month after the last immunization mice were challenged on the contralateral ear via sand fly bites—10 *L. longipalpis* sand flies carrying mature *L. major* infections were used per animal. Two weeks later animals were euthanized and their ears collected and processed. Parasite burdens were determined by limiting dilution (Fig. 5F) and T cell cytokine secretion was analysed by flow cytometry after an overnight culture of total ear cells in the presence of *L. major* SLA, and additionally PMA, Ionomycin and Brefeldin A during the last 4 h of culture. For each animal, including controls, parasite burden values were plotted against the frequencies of CD4 + T cells secreting IFN-γ, IL-10 or IL-4, in a way to assess their correlation (**A**). These frequencies are represented for VR-2001-SfSPChimera immunized and control animals, sub-grouped (Fig. 5F) according to parasite burden values (**B**). IFN-γ/IL-10 and IFN-γ/IL-4 ratios were calculated and are represented using the same group division criteria (**C**). The correlation between these ratio values and the parasite burdens was also assessed, now only considering the animals immunized with VR-2001-SfSPChimera DNA vaccine (**C**). Results from two independent experiments are represented. Each dot represents one animal. Average and SD of the values within each group are shown. Statistical differences are properly identified (Mann–Whitney test: *p ≤ 0.05 and **p ≤ 0.01) and refer to differences between sub-groups (black bars), or differences between VR-2001-SfSPChimera vaccinated and control groups (grey bars). Pearson correlation coefficients (r) and significance are also represented.

### Protection from sand fly-transmitted infection is dependent on high IFN-γ/IL-10 and IFN-γ/IL-4 ratios

The aforementioned immunogenic potential of the engineered chimeric antigen that indicates a potential “vaccine-spectrum enlargement”, led us to conduct a preliminary efficacy trial in the context of vector transmitted infections. First, we explored the efficacy of our chimeric DNA vaccine in the context of cutaneous leishmaniasis, using C57BL/6 mice, the murine model that parallels human disease. Briefly, mice were immunized intradermally in the right ear, three times at 2 week intervals with 5 µg of either VR-2001-SfSPChimera or the empty vector, and challenged 1 month later on the contralateral ear via sand fly bites: 10 *P. duboscqi* sand flies (per animal) carrying mature *L. major* infections (Fig. 5A). Ear lesion progression was assessed weekly, and total parasite burdens were evaluated at 2 and 6 weeks post challenge (Fig. 5A). In this context, no differences were detected between vaccinated and control animals, considering both pathology, assessed by ear thickness measurements (Fig. 5B), and total ear parasite burdens determined at the 2 and 6 week time-points (Fig. 5C). These results dictated the use of a different model. For that, the overall experimental time-line adopted was similar, but the murine strain used was different (BALB/c), as was the vector parasite-combination (*L. longipalpis*–*L. major*, typically a VL vector, transmitting a CL causative parasite species)—Fig. 5D. In this context, lesion development was also similar comparing vaccinated with control animals up to week 4 post-challenge (Fig. 5E). From then on, the average lesion diameter of control animals increased in a higher proportion comparing with animals immunized with VR-2001-SfSPChimera, though the differences were not statistically significant. These results aligned with the total ear parasite burdens determined. While at 2 weeks post-challenge no differences were noted, at 6 weeks post-infection the VR-2001-SfSPChimera vaccinated animals showed significantly lower parasite burdens in comparison with the animals that received the empty vector (*p* ≤ 0.05; Fig. 5E).

Considering that the phenotype observed at 6 weeks post-infection in BALB/c mice must be a consequence of an immune response initiated earlier, we decided to look at local anti-*Leishmania* immune responses early after infection (2 weeks’ time-point) using an ex vivo approach. Briefly, cytokine secretion by ear CD4 + T cells was evaluated after an overnight culture of total ear cells in the presence of SLA*.* Since at 2 weeks post-challenge the determined parasite burdens were similar, comparing vaccinated with control animals, the first step of our analysis was the correlation of those parasite burdens with the respective *Leishmania*-specific cytokine responses. Curiously, while no correlation was observed between *Leishmania*-specific IFN-γ secretion by CD4 + T cells and parasite burdens, a positive correlation was defined between parasite burdens and both *Leishmania*-specific IL-4 and IL-10 secretion by CD4 + T cells (*p* ≤ 0.05; Fig. 6A). To understand if this correlation was a result of the natural heterogeneity of our in vivo model, or a consequence of a vaccine-induced effect, we further investigated these *Leishmania*-specific cytokine responses, now in the context of a divergent analysis. For this we divided both vaccinated and control animals in two sub-groups (total of 4 groups)—the ones with determined parasite burdens below average, and the above average counterparts (Fig. 5F). This analysis revealed first that the *Leishmania*-specific IFN-γ levels did not differ comparing the sub-groups, considering both animals that received VR-2001-SfSPChimera and the ones that received the empty vector, and were significantly higher comparing all the vaccinated animals with all the controls (*p* ≤ 0.01; Fig. 6B). Importantly, for both IL-4 and IL-10 secretion by CD4 + T cells in response to *Leishmania* antigens, the picture was different. While no differences were detected comparing all the VR-2001-SfSPChimera vaccinated animals with all the controls, when we looked at sub-groups, no differences were detected within the controls, while a significant higher frequency of *Leishmania*-specific IL-4 and IL-10 secreting CD4 + T cells was detected comparing the vaccinated animals showing parasite burdens above average, with the ones with determined parasite burdens below average (*p* ≤ 0.05; Fig. 6B). Consequently, when we looked at the pro/anti-inflammatory cytokine ratios, evident differences were observed, only within the VR-2001-SfSPChimera vaccinated sub-groups (Fig. 6C). Considering both IFN-γ/IL-10 and IFN-γ/IL-4 ratios, they were significantly higher in the vaccinated animals with determined parasite burdens below average, in comparison with the ones showing parasite burdens above average (*p* ≤ 0.05 and *p* ≤ 0.01, respectively) (Fig. 6C). Indeed, when we correlated these ratios with parasite burdens, now only for the VR-2001-SfSPChimera immunized animals, robust negative correlations were established (*p* ≤ 0.01/r = −0.8649 and *p* ≤ 0.05/r = −0.7494, respectively).

## Discussion

The quest for a vaccine against human leishmaniasis has still not been fruitful. Although many vaccine candidates were proposed and tested along the years, so far, the documented protection as a result of leishmanization campaigns was never again achieved in the clinical context^12^. There are a number of factors that together justify this fact, one of the most important being related to pre-clinical to clinical translation. The majority of the vaccine candidates are initially tested empirically, as proof-of concept studies in mouse models^12^, recognized by the scientific community as invaluable tools for the advancement of science in general^30,31^. Still, there are well known differences between mouse and human immunology that may condition distinct vaccination outcomes, particularly when we consider T cell vaccines. T cell epitopes are usually HLA-restricted, frequently in a species specific fashion^32,33^. Additionally, together with the intrinsic physiological/biological/genetic limitations of murine models^30,32–34^, again having in mind translation to humans, we also need to consider the validity of the model chosen^35^. Most of the proposed anti-*Leishmania* vaccines were evaluated in models that disregarded either the contribution of the vector and vector derived components, or the transmission mechanism per se. On the one hand, the parasites are deposited into the host skin through the bite of an infected sand fly. Therefore, the early immune response in the skin must be considered and not only from the pathophysiological perspective. However, this is frequently overlooked, particularly when we think about visceral disease models that are often infected intravenously or intraperitoneally^36^. On the other hand, the parasites are inoculated at the bite site together not only with parasite-derived components (such as exosomes or PSG) but also with vector-derived ones (such as sand fly midgut microbiota and sand fly saliva), all demonstrated to impact parasite establishment^37–41^. Still, the majority of the inoculums used in experimental infections are composed exclusively of parasites (not rarely non-metacyclic promastigotes, the natural infectious form)^36,42^. Curiously, and in line with these raised issues, Peters et al.have shown in vivo that a previously defined effective anti-*Leishmania* vaccine, lost its protective potential in the context of natural *Leishmania* transmission via sand fly bites^43^, reproducing the disappointing results obtained in human clinical trials.

The above-mentioned major issues of the anti-*Leishmania* vaccines’ research were the premise of this study. The demonstration in different models that pre-exposure to vector saliva, at least from *P. papatasi*, *P. duboscqi*, *L. whitmani* or *L. longipalpis*, confers protection against vector-transmitted leishmaniasis^13,18,44,45^, supports the study of defined sand fly salivary proteins as anti-*Leishmania* vaccines. Importantly, the fact that the protection is mediated by specific, but indirect non-parasite specific responses, can be considered an advantage if we think of a paracrine effect mediated by non-infected antigen-presenting cells^46^ that are potentially less affected by the known *Leishmania* immunomodulatory potential^3^. However, using a sand fly saliva-based vaccine presents its own challenges. The natural evolutionary pressure that conditioned the speciation of the different Phlebotominae that transmit leishmaniasis^47^, brought as a logical consequence the lower potential for cross-reactivity (due to the known—although not strict—relation between T cell epitopes’ conservation and immunogenicity^48^). Therefore defined sand fly salivary proteins will have a limited anti-*Leishmania* vaccine spectrum: effectiveness will probably be limited to the geographical region where the vector species is prevalent, and consequently to the parasite species and disease manifestation associated to that specific vector^21,22^. In the hope of turning limitations into opportunities, we engineered a single antigen (vector-derived) composed of fractions of two distinct sand fly salivary vaccine candidates, through a reverse vaccinology approach that potentially maximizes translation from the pre-clinical to the clinical context, which we propose as a proof of principle of a future vector-based pan-*Leishmania* vaccine.

Although our vaccine proposal is directed to humans, in our in silico approach, we predicted, both murine and human MHC-I and MHC-II restricted epitopes (Figs. 1, 2), to uncover the “immunogenic map” of the two antigens and the overlaps considering the two species. This approach was adopted to bridge the pre-clinical and clinical contexts. Peptide-based vaccines, particularly targeting T cell responses, have been exploited particularly in the cancer immunotherapy field, and have recognized advantages pertaining to simple and reproducible manufacturing processes, stability under simple storage conditions, and lower probability of generating allergic or autoimmune responses^49^. However, peptide vaccines are also recognized as poor immunogens, with a limited spectrum considering the multiplicity of MHC alleles of the human population, and a higher probability of in vivo degradation in comparison with folded proteins^49^. Therefore, instead of considering individual peptides in our convergent analysis, we opted to select highly immunogenic protein regions. One the one hand this enriches epitope diversity and ensures “natural” antigen processing and presentation and on the other allows for the potential targeting of a wider population. Indeed, the protein portions we have selected have the potential of pan-immunogenicity in humans, containing epitopes predicted to bind to 24/27 and 20/27 MHC-II and MHC-I human alleles, respectively in the case of PdSP15, and to 27/27 of both MHC-II and MHC-I human alleles in the case of LJL-143 (having in mind the MHC-II and MHC-I reference sets used provide ˃ 99% and ˃ 97% population coverage, respectively). Still, in silico can never replace in vitro/in vivo observations, and must always be performed rationally. We included, on purpose, the signal peptide sequences of both proteins in our predictions and analysis, and found these are indeed highly immunogenic (considering both murine and human MHC-I and MHC-II alleles), which goes in line with data published on cancer research and *M. tuberculosis* infection^50,51^. However, if in other contexts they can be considered as potential vaccine candidates (they are expected to be on the diverse pool of presented peptides in the context of bacterial infections or cancer)^50^, this is virtually impossible when we think of sand fly-saliva based vaccines: in nature, the host immune system will only “see” the mature salivary proteins (that are deposited in the skin by the bite of the vector), and never the signal peptides that will remain within the insect salivary glands secretory cells.

One may argue that to simply fuse the sand fly salivary proteins in tandem, would be an equally valid approach, and perhaps easier or at least faster, considering the time the in silico predictions and rational analysis consumes. However, sand fly salivary proteins are recognized by their strong bioactive properties^23^. Indeed the two proteins used in this study have strong anti-haemostatic properties. LJL-143 is an anti-coagulant that acts through the inhibition of factor Xa of the coagulation cascade^28^, and PdSP15 interferes with factor XII contact-system driven clotting^27^. Once the mere fusion of two proteins might not impact their bioactive properties (there are actually therapeutic approaches in the pre-clinical stage, as well as biotechnology strategies that depend on the maintenance of bioactive properties in the context of fusion proteins^52–54^) we opted to fuse the immunogenic portions of the two proteins in an interpolated manner (Fig. 3), as a way to potentially destabilize protein structures and consequently eliminate the bioactive effects, thereby generating an innocuous antigen. Of note, to maximize the potential loss of protein bioactive potential we considered the protein residues thought to be important for their biological activities in the in silico convergent analysis, excluding them, at least partially from the selected protein portions, while assuring the maintenance of potential broad-spectrum immunogenicity. The same rationale is valid if we consider a multivalent vaccine composed by different salivary antigens (for instance the two recombinant salivary proteins formulated together), with an additional drawback. Multivalent vaccines are associated with higher costs of production, a consequence of manufacturing costs increasing proportionally to the number of antigens contemplated, and the necessary application of exhaustive quality control measures^55^, which is a limitation when we think of vaccine development for leishmaniasis, a neglected tropical disease (NTD). NTDs funding averages a total of 0.6% of total development assistance for health^56^, although 1/6 of the human population suffer from at least one^57^—for the sake of comparison average funding for HIV averages a total of 36.3% of total development assistance for health (60 fold higher)^56^, while the estimated prevalence among adults is around 0.8% (20 fold lower)^58^.

To validate our in silico analysis we opted to perform recall experiments against sand fly saliva and not against the single recombinant proteins, to mimic better the natural infection context where a heterogeneous mixture of salivary proteins is inoculated into the host skin when the sand fly takes a blood meal. T cell responses, particularly activation, are known to be dependent, amongst other things, on antigen dose, relevant not only in the context of primary immune responses^59,60^, but also in relation to memory responses^61,62^. Given the fact that around 500 ng representing more than 10 different salivary proteins described in distinct species, dependent on species-specific differences in their relative abundance, are deposited in host skin during a sand fly blood meal^63^, it’s not unreasonable to consider that some may not be relevant enough to reach the antigen threshold requirement for T cell proliferation, translating into non-immunogenicity. Remarkably, we demonstrate that T cells from mice immunized with VR-2001-SfSPChimera, expand in response to both *P. duboscqi* and *L. longipalpis* SGH (Fig. 4), a result that validates our in silico predictions, and attests our hypothesis of vaccine-spectrum enlargement. These results, considering our convergent in silico analysis contemplating both murine and human MHC alleles, suggest that this fusion protein will potentially generate relevant responses both in individuals from South America and West Africa (*L. longipalpis* and *P. duboscqi* origins, respectively), hypothesis we are currently pursuing. Of note, T cells from the positive control groups immunized with *P. duboscqi* or *L. longipalpis* SGH did not respond to the heterologous SGH stimulus, (*L. longipalpis* or *P. duboscqi* SGH, respectively) indicating an absence of cross-reactivity and therefore pointing to the potential limited spectrum of sand fly salivary antigens.

Although we clearly demonstrate the vaccine spectrum enlargement with respect to immunogenicity, we were not able to conclude the same regarding efficacy. When we assessed protection in the context of *L. major* transmission by *P. duboscqi* to C57BL/6 mice, a self-healing cutaneous leishmaniasis model that parallels best human disease, we did not observe any protection in animals that received the VR-2001-SfSPChimera DNA vaccine (Fig. 5B,C). In this particular infection context, we could mainly evaluate how the memory generated by the PdSP15 antigenic portions included in the fusion vaccine would influence parasite establishment (since the recall agent to consider is the native PdSP15 salivary protein from *P. duboscqi* sand flies). Importantly, this protein (either in the context of DNA vaccine, or as recombinant antigen) was never tested as an anti-*Leishmania* vaccine in murine models of cutaneous leishmaniasis, although it was demonstrated an effective vaccine in a Non-Human Primate model^18^. Still, a closely related homologue from *P. papatasi* (PpSP15; Supplementary Figure 3; 67% identity considering the full length protein), was demonstrated to protect C57BL/6 mice infected intradermally by needle with *L. major* metacyclic promastigotes together with *P. papatasi* SGH^17^. Curiously, our in silico analysis of the PdSP15 protein did not show any relevant MHC-II restricted epitopes with respect to the allele relevant for C57BL/6 background (Supplementary Data 2), making us wonder if the efficacy results would be different, for instance in the context of a NHP model (retaining the reported protective effect of the full length PdSP15 antigen^18^), or even considering in humans where in silico predictions suggest a good immunogenic potential. Importantly, our predictions align with the fact that PBMCs from individuals living in central Mali, where *P. duboscqi* sand flies are prevalent, were demonstrated to respond to a stimulation with PdSP15 recombinant protein and secrete IFN-γ (and not only) in a significant fashion, the cytokine associated with the protective phenotype observed in non-human primates.

On the other hand, when we assessed the potential effect of the immune response elicited by the LJL-143 immunogenic portions included in the engineered fusion antigen on parasites’ establishment, we observed a modest, but significant effect 6 weeks post-infection (Fig. 5E,F). For this, we took advantage of *L. longipalpis* sand flies (main vector of VL and South America, containing the native LJL-143 in the saliva) and BALB/c mice (one of the relevant murine models of VL), but in the context of *L. major* transmission (dermotropic parasites). Curiously, this is the first time that protection mediated by LJL-143 is demonstrated in a murine model. Although this antigen was demonstrated to negatively impact *Leishmania* parasites in a relevant canine model, since dogs are natural reservoirs of VL^25^, in a different murine model (C57BL/6 mice infected intradermally by needle with *L. major* parasites together with *L. longipalpis* SGH), LJL-143 failed to protect against disease^64^, emphasizing the importance of host genetics. This different phenotype we report now, may be due to two different explanations. The first one relates directly to the model. Again, the in silico predictions indicate that this antigen is theoretically more immunogenic in the BALB/c background (Supplementary Data 8—various MHC-II restricted epitopes in at least 4 distinct protein regions), comparing with C57BL/6 mice (Supplementary Data 9—6 MHC-II restricted epitopes in one single regions), which may suggest that the absence of efficacy in the C57BL/6 model may be consequence of poor immunogenicity (although a LJL-143 specific DTH response was reported in this background^64^). The second one comes as an (accidental) consequence of our strategy. In silico, we predicted MHC-I and MHC-II restricted epitopes. Still, this analysis does not allow to extrapolate to T cell functionality that has to be proven experimentally, strategy that is starting to be employed in T cell vaccinology^65^. It is therefore not impossible to imagine that, by removing 26% of the LJL-143 protein sequence (portions not included in our fusion antigen), we excluded important Th2 epitopes, overall improving the protective potential of the antigen. This is however a speculation that we think deserves further investigation.

The curious observation that the protective phenotype in BALB/c mice was observed starting only from week 4 post-infection (and not at the 2 weeks’ time-point) led us to further explore the potential mechanism of protection. Since the protective mechanism in our context has to be dependent on the anti-vector saliva response, differences must be visible relatively early after vector transmission (the moment where the host gets in contact with vector saliva), which justifies our option to explore the immune context at 2 weeks post-infection (where no differences in terms of both pathology and parasite burdens were observed). Because the dispersion in terms of the quantified parasite burdens (at 2 weeks post-infection) might just be a consequence of the heterogeneity of sand fly transmission experiments, we divided both controls and VR-2001-SfSPChimera vaccinated animals in two subgroups, and showed that indeed the phenotype observed at 6 weeks post-infection is vaccine specific (Fig. 6). Importantly, we show here that a strong IFN-γ response by CD4 + T cells, accepted as the hallmark protective response^66^, is not sufficient to warrant protection. In fact, the best correlates of protection in our model are the low *Leishmania*-specific secretion of the anti-inflammatory cytokines IL-4 and IL-10 by CD4 + T cells, and consequently the high anti/pro-inflammatory balance. The next step we will follow will be the evaluation of the vaccine candidate in the context of a visceral disease model (e.g. *L. infantum* transmission by *L. longipalpis*, the natural parasite-vector pairing from South America), with a certain degree of optimism, since also in visceral disease, a high IFN-γ response, together with a lower IL-10 response are essential for parasite elimination and disease resolution^67,68^.

In summary, we show here that the enlargement of sand fly saliva-based vaccines is an achievable goal. This should however be done following a rational strategy, to ensure that the probability of translatability to humans, which are the target population, is the highest possible. We propose our approach as a proof of principle for a future vector-based pan-*Leishmania* vaccine encompassing immunogenic portions from more salivary proteins, selected based on a convergent analysis contemplating murine and human MHC alleles. For example, salivary proteins from *P. argentipes* or *P. sergenti*, relevant vectors of visceral and cutaneous disease, caused by *L. donovani* and *L. tropica*, in the Indian Sub-continent and Middle East, respectively^69,70^ can be considered, to further enlarge the vaccine-spectrum of the present *P. duboscqi* and *L. longipalpis* fusion protein. There is still much room for improvement, starting by switching from DNA to a recombinant protein vaccine approach that can be administered together with a Th1 inducing adjuvant and/or combined with a parasite-derived highly conserved antigen, to maximize the negative impact on *Leishmania* parasites’ establishment, through additive parasite-specific and vector saliva-specific immune responses.

## Methods

### Rationale

As a proof-of principle that would help us understand if we could obtain a vector-derived large-spectrum anti-*Leishmania* vaccine candidate for humans (targeting distinct endemic areas, in both the New and Old Worlds; directed against different parasite species, and consequently distinct disease forms), we have selected two different sand fly salivary proteins, already studied in the context of vector-based anti-*Leishmania* vaccines, and followed a reverse vaccinology approach to design a single antigen (fusion protein) that in theory would maintain the individual immunogenic properties of each of the single sand fly salivary proteins, but not their biological activities. The proteins this study focuses on are: (1) PdSP15, a *P. duboscqi* salivary protein (Old World; studied in the context of Cutaneous Leishmaniasis)—GenBank Acc. No. ABI15933^71^; and (2) LJL-143, a *L. longipalpis* salivary protein (New World; studied in the context of Visceral Leishmaniasis)—GenBank Acc. No. AAS05319^72^.

### In silico analysis

Signal peptide sequences were predicted using the SignalP 3.0 server (https://www.cbs.dtu.dk/services/SignalP-3.0/)^73^. T cell epitope mapping was performed (for each individual protein) using the IEDB Analysis Resource (www.iedb.org). MHC-II binding predictions were made against the only 3 available murine alleles (H2-IA^d^ and H2-IE^d^—representative, among others , of the BALB/c strain; and H2-IE^b^—representative, among others, of the C57BL/6 strain), and 27 human alleles (reference set that provides ˃ 99% population coverage^74,75^), considering 15-MER sized peptides, and using the consensus method^76,77^, or whenever not possible the SMM-Align (ver. 1.1)^78^ or the NetMHCIIpan (ver. 3.1)^79^ tools. MHC-I binding predictions were made against the only 5 available murine alleles (H2-D^d^, H2-K^d^ and H2-L^d^—representative, among others, of the BALB/c strain; and H2-D^b^ and H2-K^b^—representative, among others, of the C57BL/6 strain), and 27 human alleles (reference set that provides ˃9 7% population coverage^74,80^), considering 8–14- or 9–10-MER sized peptides, respectively, and using the consensus tool^81^, which combines predictions from ANN aka NetMHC (4.0)^82–84^, SMM^85^ and Comblib^86^ tools, or whenever not possible the NetMHC (4.0) tool^82–84^. Additionally, human CD4 + T cell epitope mapping (MHC-II binding against the same 27 alleles that provide ˃  99% population coverage), was performed with two other salivary proteins: PpSP15—GenBank acc. no. AF335487—a *Phlebotomus papatasi* salivary protein with known homology to PdSP15, studied in the context of Cutaneous Leishmaniasis^63,87^; and PpeSP06—GenBank acc. no. DQ153100—a salivary protein from *Phlebotomus perniciosus*, the main vector of Visceral Leishmaniasis in the Mediterranean Basin, with known homology to LJL-143^87,88^. Selection of predicted binders was done based on the percentile ranks calculated, according to IEDB recommendations: top 10% or 1% hits for MHC-II or MHC-I, respectively^89^. A convergent analysis was performed to identify the protein portions to be part of the final fusion vaccine candidate based on: (1) presence of murine and human CD4 + T cell epitopes (predicted); (2) presence of murine and human CD8 + T cell epitopes (predicted); (3) conservation of protein (and epitopes) sequences between homologs; and (4) exclusion of signal peptides and other residues thought to be important for the proteins’ biological activities.

### Construction of the multiepitope vaccine sequence, cloning and other in-silico determinations

The sequences coding for the protein portions selected to be included in the fusion antigen were joined contiguously, respecting the open reading frames of interest. Sequences coding for a Flag-Tag and a His-Tag were added to the 3′ end, followed by the introduction of a stop codon. Correct translation was confirmed using the ExPASy translate tool (https://web.expasy.org/translate/), followed by codon optimization to a mammalian codon usage bias. The originated sequence was synthetized (GeneArt Strings—Thermo Fischer Scientific, MA, USA) and cloned into the VR2001-TOPO vector as previously described^29^. The VR-2001-SfSPChimera plasmid was then purified as previously reported^29^, and used either as a DNA vaccine or as a vector in the attempt of protein expression using a mammalian system, described elsewhere^25^.

Additional *in-silico* determinations were performed now using the multiepitope antigen protein sequence as template. The prediction of antigenicity probability (ANTIGENpro), solubility upon overexpression (SOLpro), formation of disulphide bonds (DIpro) and presence of transmembrane domains (ABTMpro) were performed using the SCRATCH protein predictor online tool (https://scratch.proteomics.ics.uci.edu/). The prediction of allergenic potential was performed using the AlgPred online tool (https://crdd.osdd.net/raghava/algpred/)^90^.

### Ethics statement

All animal experimentation was carried out in accordance with i) the Portuguese National Authorities for Animal Health guidelines, according to the statements on the Directive 2010/63/EU of the European Parliament and of the Council; ii) and the Guide for the Care and Use of Laboratory Animals, in line with the NIH Office of Animal Care and Use and Animal Research Advisory Committee guidelines. The i3S Animal Ethics Committee and the National Institute of Allergy and Infectious Diseases (NIAID) Animal Care and Use Committee (under the animal protocol LMVR4E) approved all the experiments.

### Mice

Six- to eight-week-old female BALB/c and C57BL/6 mice were obtained from Charles River laboratories. Animals were maintained (1) under specific pathogen-free conditions at the i3S facilities, in sterile IVC cabinets, with food and water available ad libitum; or (2) were housed under pathogen-free conditions at the NIAID Twinbrook animal facility, Rockville, MD. Animals were randomly assigned to groups.

### Sand flies and salivary glands homogenates (SGH)

*Phlebotomus duboscqi*, *L. longipalpis*, *P. papatasi* and *P. perniciosus* sand flies were mass reared at the Laboratory of Malaria and Vector Research insectary according to the protocols described elsewhere^91^. Adult females were maintained on a 30% sucrose diet (commercial sugar) and were starved for 12 h before feeding. Three- to 4-day-old sand flies were used for transmission experiments. Five- to 7-day-old sand flies were used for preparation of SGH. Briefly, pools of 20 salivary glands were dissected in phosphate-buffered saline, subjected to ultra-sonication followed by centrifugation at 10,000*g* for 3 min at 4 °C. The supernatant (SGH) was then collected and stored at − 80 °C until use.

### Parasites and soluble leishmania antigen (SLA)

A cloned line of virulent *Leishmania major* (WR 2885) isolated from a soldier deployed to Iraq^16^ was used in this study. Promastigotes were routinely maintained at 26 °C in Schneider’s insect medium (Lonza, MD, USA) supplemented with 10% heat inactivated foetal bovine serum (Thermo Fischer Scientific, MA, USA), penicillin (100 U/ml) and streptomycin (100 mg/ml). SLA was prepared from 1 × 10^9^ *L. major* promastigotes. Briefly, parasites in PBS were subjected to repeated freeze–thaw cycles, and centrifuged at 10,000*g* for 20 min at 4 °C. The resultant supernatant (SLA) was collected, and its protein content was quantified by the BCA method. Aliquots (1 mg/ml) were prepared and frozen at − 80 °C until use.

### Immunization scheme

Mice were immunized intradermally in the right ear three times at 2 weeks intervals with 5 µg of VR-2001-SfSPChimera plasmid in 10 µl PBS. Negative controls received either the same amount of empty vector (VR-2001 in 10 µl PBS; used as “true placebo” in efficacy trials, according to the WHO recommendations^92^) or the same volume of vehicle only (10 µl PBS). Additionally, for immunogenicity ex vivo experiments, two additional control groups (positive controls) were immunized intradermally with either *P. duboscqi* or *L. longipalpis* SGH—the equivalent of 1 sand fly salivary gland pairs in 10 µl PBS. One month after the last immunization, animals were either euthanized for vaccine immunogenic potential determination (and bioinformatics validation) or challenged via sand fly bites.

### Transmission of *L. major * via sand fly bites

*P. duboscqi* or *L. longipalpis* sand flies were infected by artificial feeding through a chick membrane on defibrinated rabbit blood (Spring Valley Laboratories, MD, USA) containing *L. major* promastigotes, as previously described^16^. Fully blood-fed female sand flies were separated and maintained at 26 °C with 75% humidity and were provided 30% sucrose until the development of a mature infection was confirmed (typically 11–15 days after blood feeding). Sugar pads were removed 12 h before transmission. Ten *L. major*-infected sand flies were applied to the left ear of each mouse (under anaesthesia, as previously described^39^), using vials with a meshed surface held in place by custom-made clamps, and allowed to feed for two hours in the dark (26 °C with 75% humidity). The number of blood-fed flies was then determined by observing them under a stereomicroscope, as a potential measurement of transmission success.

### Post-infection follow up and experimental endpoints

Animals were monitored to evaluate the evolution of the characteristic lesions caused by *L. major* infection. Ear thickness or the maximum lesion diameter were measured individually using a Digimatic calliper (Mitutoyo Corp., IL, USA) and recorded on a weekly basis. Animals were euthanized at 2- or 6-weeks post-infection for parasite burden determination, as well as for the dissection of potential correlates of protection (2-weeks’ time-point only).

### Euthanasia, organ collection and manipulation

Euthanasia was performed by cervical dislocation always under isoflurane anaesthesia (Piramal healthcare, UK). Spleens were aseptically collected from euthanized animals, weighed, and homogenized using Falcon 100 µm Cell Strainers (Corning Life Sciences, MA, USA). Ears were collected, immersed in 70% ethanol and then in PBS. Ear cell suspensions were obtained as previously described^43^. Briefly, the ventral and dorsal sheets were separated, deposited in RPMI containing 0.25 mg/ml Liberase CI purified enzyme blend and 10 µg/ml DNAse (both from Roche, Switzerland), and incubated for 1 h at 37 °C and 5% CO_2_. Digested ear sheets were subsequently homogenized for 3 min using the Medicon/Medimachine tissue homogenizer system (BD, NJ, USA) and the resultant single cell suspensions were filtered using a 70 µm-pore size Falcon cell strainer (BD Biosciences, NJ, USA). Viable cell numbers were determined for all obtained cell suspensions (ear and spleen derived) under the microscope, using a Neubauer chamber and trypan blue solution as the “viability marker”.

### Ex vivo splenic cell proliferation assays

Stimulations were performed with antigen-pulsed Bone Marrow-derived Dendritic Cells (BMDCs), differentiated from naïve strain-matched mice bone marrow precursors as previously described^40^. BMDCs (5 × 10^4^/well) were pulsed at day − 1 with *P. duboscqi* or *L. longipalpis* or *P. papatasi* or *P. perniciosus* SGH (final concentration of 3 sand fly salivary gland pair/ml). Non-pulsed cells were cultured in parallel to be used in the experimental-control conditions. Splenic cell suspensions were aseptically pre-stained (day 0) as previously described^26^. Briefly, 1 × 10^7^ splenocytes per animal were pelleted, washed twice with PBS and stained for 10 min at 37 °C with CFSE (1 μM; in 1 ml of PBS; Thermo Fisher Scientific, MA, USA). Complete RPMI (Lonza, Switzerland) [10% heat-inactivated FBS (Lonza, Switzerland), 2 mM l-glutamine, 100 U/ml penicillin and 100 μg/ml streptomycin (BioWhittaker, MD, USA)] was added to the cell suspensions, that were then pelleted, re-suspended in complete RPMI and incubated at 4 °C for 5 min to stop the reaction. Afterwards, cells were once more centrifuged (5 min, 350 g), and resuspended in complete RPMI supplemented with 50 μM 2-mercaptoethanol (Sigma-Aldrich, MO, USA). Stained splenocytes were added to the pulsed/non-pulsed BMDCs at the final amount of 2.5 × 10^5^ cells per well (1:5—DC:Splenocyte ratio). Additionally, to the non-pulsed DCs + splenocytes co-cultures, concanavalin A (3 μg/ml) and complete RPMI medium were added as positive and negative controls, respectively. Co-cultures were incubated (37 °C and 5% CO_2_) for three (positive control) or 4 days (remaining conditions), and proliferating T cell populations were determined by flow cytometry. Each determination was performed in duplicate.

### Ex vivo ear cell stimulation for intracellular cytokine quantification

Ear cell suspensions (ideally at least 1 × 10^6^ cells in complete RPMI) were seeded in 24-well plates and cultured for 18 h in the presence of 50 µg/ml of SLA at 37 °C and 5% CO_2_. PMA, Ionomycin and Brefeldin A (BD Biosciences, NJ, USA) were added during the last 4 h of culture at the concentrations of 25 ng/ml, 500 ng/ml and 10 µg/ml, respectively. Cells were then harvested and T cell cytokine production was analysed by flow cytometry.

### Flow cytometry

The anti-mouse monoclonal antibodies used to perform this study were either purchased to BD Biosciences (BD—NJ, USA) or BioLegend (BioL—CA, USA): FITC labelled anti-CD4 (GK1.5, BD); PE labelled anti-IFNγ (XMG1.2, BD); PerCP-Cy5.5 labelled anti-CD8α (53–6.7, BD); PE-Cy7 labelled anti-CD3 (HA2, BioL), and anti-IL-10 (JES5-16E3, BD); APC labelled anti-IL-4 (11B11, BD); BV421 labelled anti-TGFβ1 (TW7-16B4, BD); BV510 labelled anti-CD4 (RM4-5, BioL); and APC/Fire labelled anti-TCRβ (H57-597, BD). Cell viability was evaluated using either the LIVE/DEAD Fixable Yellow Dead Cell Stain Kit (Thermo Fischer Scientific, MA, USA) or the Zombie NIR Fixable Viability Kit (BioLegend, CA, USA). The viability stainings were performed first, according to the manufacturers’ instructions. Afterwards, the cells were blocked with anti-CD16/CD32 (BD Fc block, 2.4G2; BD Pharmingen) for 15 min at 4 °C. Surface stainings of splenic cells were performed in PBS + 0.5% BSA (30 min, 4°C) followed by a 15 min’ fixation step using 1% PFA. Surface stainings of ear cells were performed for 30 min at 4° C in Cell Staining Buffer (BioLegend, CA, USA), followed by 20 min fixation using a commercial Fixation Buffer (BioLegend, CA, USA). Intracellular staining of fixed ear cells was performed for 2 h at 4 °C in Perm/Wash Buffer (BioLegend, CA, USA). Cells were always washed between each step with the respective staining buffer used (PBS + 0.5% BSA, Cell Staining Buffer or Perm/Wash Buffer). Stained cells in either PBS + 0.5% BSA or Cell Staining Buffer were then acquired in a FACSCanto (BD, NJ, USA) or a MACSQuant Analyzer 16 (Miltenyi Biotech, Germany). Data analysis was performed with the FlowJo software v10 (TreeStar, OR, USA).

An initial gate plotting FSC-A versus SSC-A was performed to exclude cell debris. Afterwards, singlets were selected by plotting FSC-A versus FSC-H and the remaining cell populations were resolved within live cells. T lymphoid cell populations were defined as CD3 +/CD4 + and CD3 +/CD8 +. Cytokine production by T cells was assessed within these two sub-populations. Proliferating T cells (total, and CD4 + or CD8 +) were defined as CFSE^int/low/neg^ (FITC channel), always comparing each condition with the respective negative control. A brief gating strategy is represented in Supplementary Figure 1.

### Parasite burdens determination

Ear parasite burdens were assessed by the limit dilution method, as reported elsewhere^93^. Briefly, defined volumes of ear cell suspensions were serially diluted in 96-well plates containing 50 µl biphasic medium—NNN medium with 10% of defibrinated rabbit blood—overlaid with 200 µl of Schneider’s insect medium (Lonza, MD, USA) supplemented with 10% heat inactivated foetal bovine serum (Thermo Fischer Scientific, MA, USA), penicillin (100 U/ml) and streptomycin (100 mg/ml), and incubated for 14 days at 26 °C. The total number of parasites per ear was calculated as previously^93^.

### Statistical analysis

Results are expressed per individual animals/samples, with a representation of the group mean value ± standard deviation. Statistical differences were analysed using GraphPad Prism software v6.01 (CA, USA). As parasite numbers in the ear of mice transmitted by infected sand flies bites do not follow a Gaussian distribution we used the Mann–Whitney test in the analysis of any transmission-related data. All of the remaining analysis were performed with parametric statistical tests: unpaired t-test or One Way ANOVA (with Tukey’s post-hoc analysis) were used to compare two or more than two groups, respectively. Bivariate correlations were calculated and given by the Pearson correlation coefficient.

## Supplementary information

Supplementary Figures.

Supplementary Information 1.

Supplementary Information 2.

Supplementary Information 3.

Supplementary Information 4.

Supplementary Information 5.

Supplementary Information 6.

Supplementary Information 7.

Supplementary Information 8.

Supplementary Information 9.

Supplementary Information 10.

Supplementary Information 11.

Supplementary Information 12.

Supplementary Information 13.

Supplementary Information 14.

## Data Availability

All relevant data are within the paper and its supplementary materials.
