# Supplementary material for: Engineering a vector-based pan-Leishmania vaccine for humans: proof of principle
Source: Sci Rep. 2020 Oct 29;10:18653. doi: 10.1038/s41598-020-75410-0 (PMC7596519; doi:10.1038/s41598-020-75410-0)
Supplement: Supplementary file 1 — Supplementary Figures. [file 41598_2020_75410_MOESM1_ESM.docx]

**SUPPLEMENTARY INFORMATION**

**Engineering a vector-based pan-*Leishmania* vaccine for humans: proof of principle**

Pedro Cecílio^1-4^, James Oristian^4^, Claudio Meneses^4^, Tiago D. Serafim^4^, Jesus G. Valenzuela^4^, Anabela Cordeiro-da-Silva^1,2,3, #^ * and Fabiano Oliveira^4, #^ *

**Authors Affiliations**

^1^ i3S - Instituto de Investigação e Inovação em Saúde, Universidade do Porto, Porto, Portugal;

^2^Parasite Disease Group, IBMC - Instituto de Biologia Molecular e Celular, Universidade do Porto, Porto, Portugal;

^3^Departamento de Ciências Biológicas, Faculdade de Farmácia da Universidade do Porto (FFUP), Porto, Portugal;

^4^Vector Molecular Biology Section, Laboratory of Malaria and Vector Research, National Institute of Allergy and Infectious Diseases, National Institutes of Health, Rockville, MD, USA;

**^#^**Senior authors contributed equally to this work.

*** Address correspondence to:**

Prof. Anabela Cordeiro da Silva

Rua Alfredo Allen, 208
4200-135 Porto, Portugal

cordeiro@ibmc.up.pt

Dr. Fabiano Oliveira

TW3 Building, Room 2E32
12735 Twinbrook Parkway
Rockville, MD 20852

loliveira@niaid.nih.gov

**Short title:** Exploring vector saliva to generate a pan-*Leishmania* vaccine

**Keywords:** *Leishmania*; Cutaneous Leishmaniasis; Visceral Leishmaniasis; Sand fly salivary proteins; Vaccine; Bioinformatics; Multi-epitope; Chimera


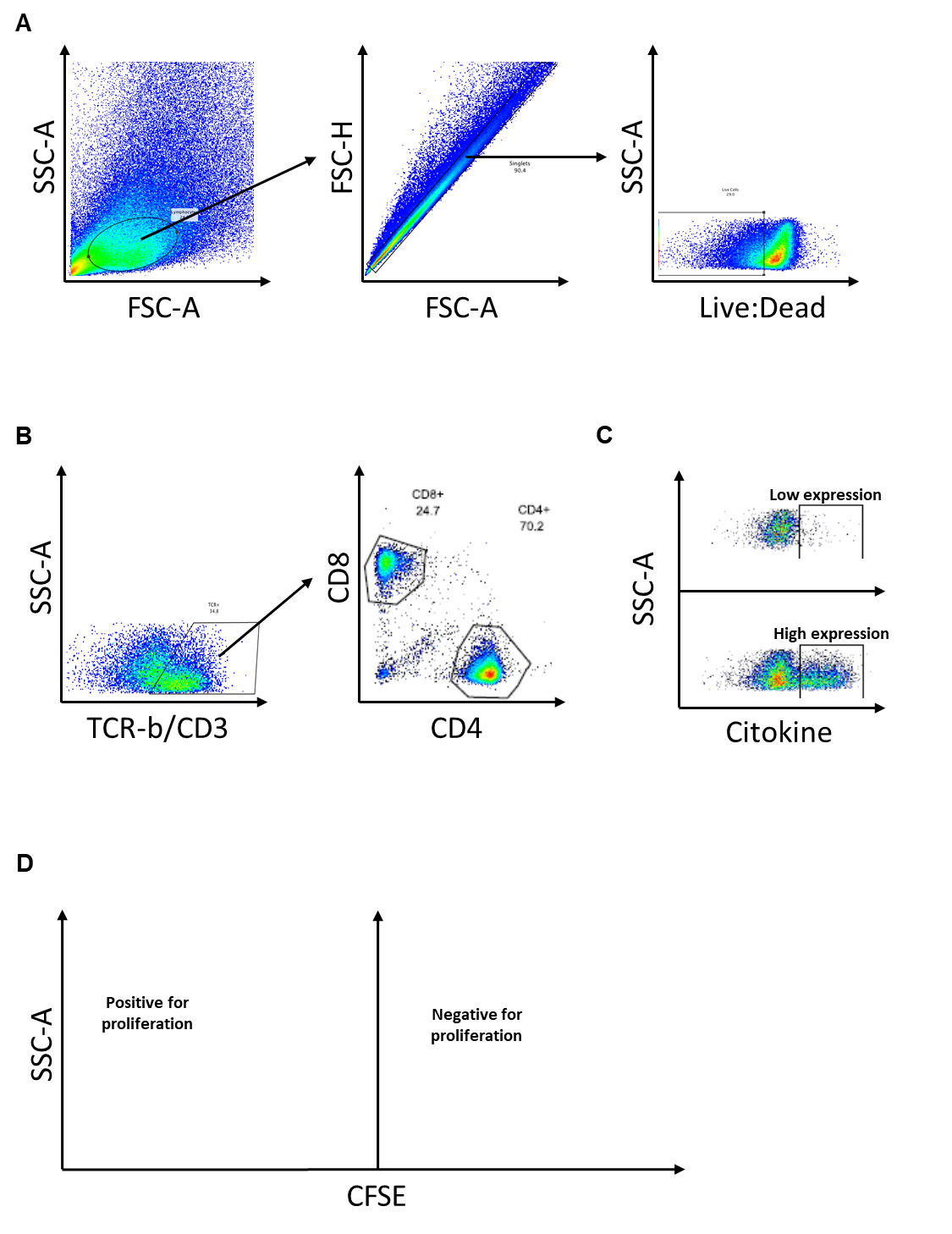


**Supplementary Figure 1. Brief representation of the flow cytometry gating strategy applied in this work. (A)** An initial gate plotting FSC-A versus SSC-A was performed to exclude cell debris and select for lymphocytes. Afterwards, singlets were selected by plotting FSC-A versus FSC-H and the remaining cell populations were resolved within live cells. (B) T lymphoid cell populations were defined as CD3+(or TCRβ+)/CD4+ and CD3+(or TCRβ+)/CD8+. (C) Cytokine production by T cells was assessed within these two sub-populations. (D) Proliferating T cells (total, and CD4+ or CD8+) were defined as CFSE^int/low/neg^ (FITC channel), always comparing each condition with the respective negative control.


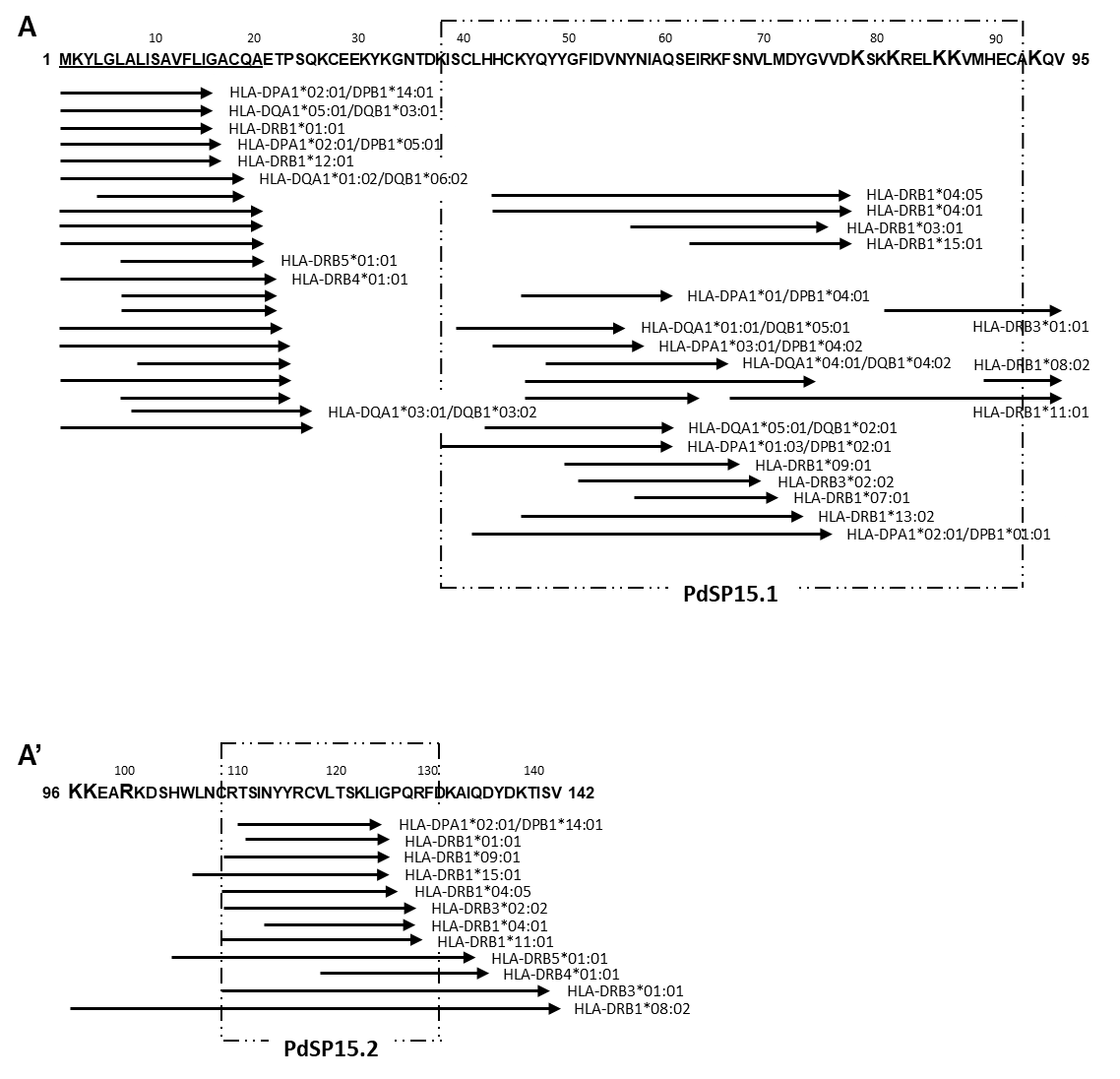


**Supplementary Figure 2. PdSP15 CD4+ T cell epitope mapping: extended representation of human MHC-II restricted epitopes.** PdSP15 (GenBank acc. no. ABI15933) CD4+ T cell epitope mapping was performed using the IEDB Analysis Resource considering 27 human MHC-II alleles. A/A’ represent the extended translation of the data set obtained (Supplementary data 5) and show predicted epitopes with percentile rank values between 2.5 and 7.5. Results are presented by allele. Each arrow represents one or more (contiguous) predicted epitopes. Underlined protein residues represent the signal peptide sequence. Magnified protein residues are potentially important for protein biological activity. Dashed boxes represent the two protein portions selected to be part of the final chimeric sand fly salivary antigen.

**
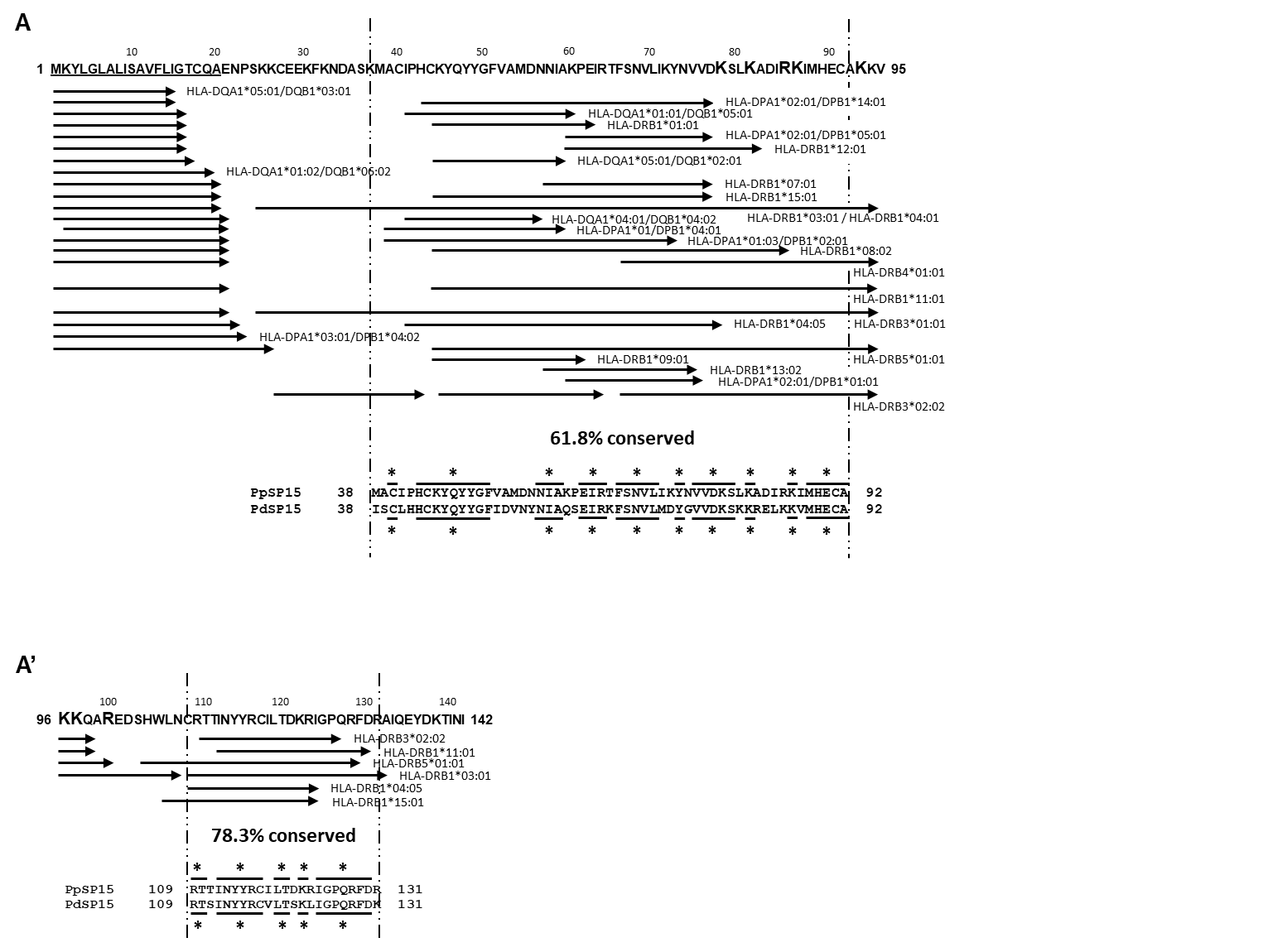
**

**Supplementary Figure 3. *in silico* determination of the human MHC-II restricted epitopes of PpSP15, a known PdSP15 homologue.** PpSP15 (GenBank acc. no. AF335487) CD4+ T cell epitope mapping was performed using the IEDB Analysis Resource considering 27 human MHC-II alleles. A/A’ represent the translation of the data set obtained (Supplementary data 6) and show the top 7.5% hits. Results are presented by allele. Each arrow represents one or more (contiguous) predicted epitopes. Underlined protein residues represent the signal peptide sequence. Magnified protein residues are potentially important for protein biological activity. Dashed vertical lines limit the homologous regions to PdSP15 portions selected to be part of the final chimeric sand fly salivary antigen. The alignment of PpSP15 and PdSP15 portions of interest is represented, with the conserved residues highlighted. Percentage of amino acid sequence conservation within each portion was calculated and is also shown.


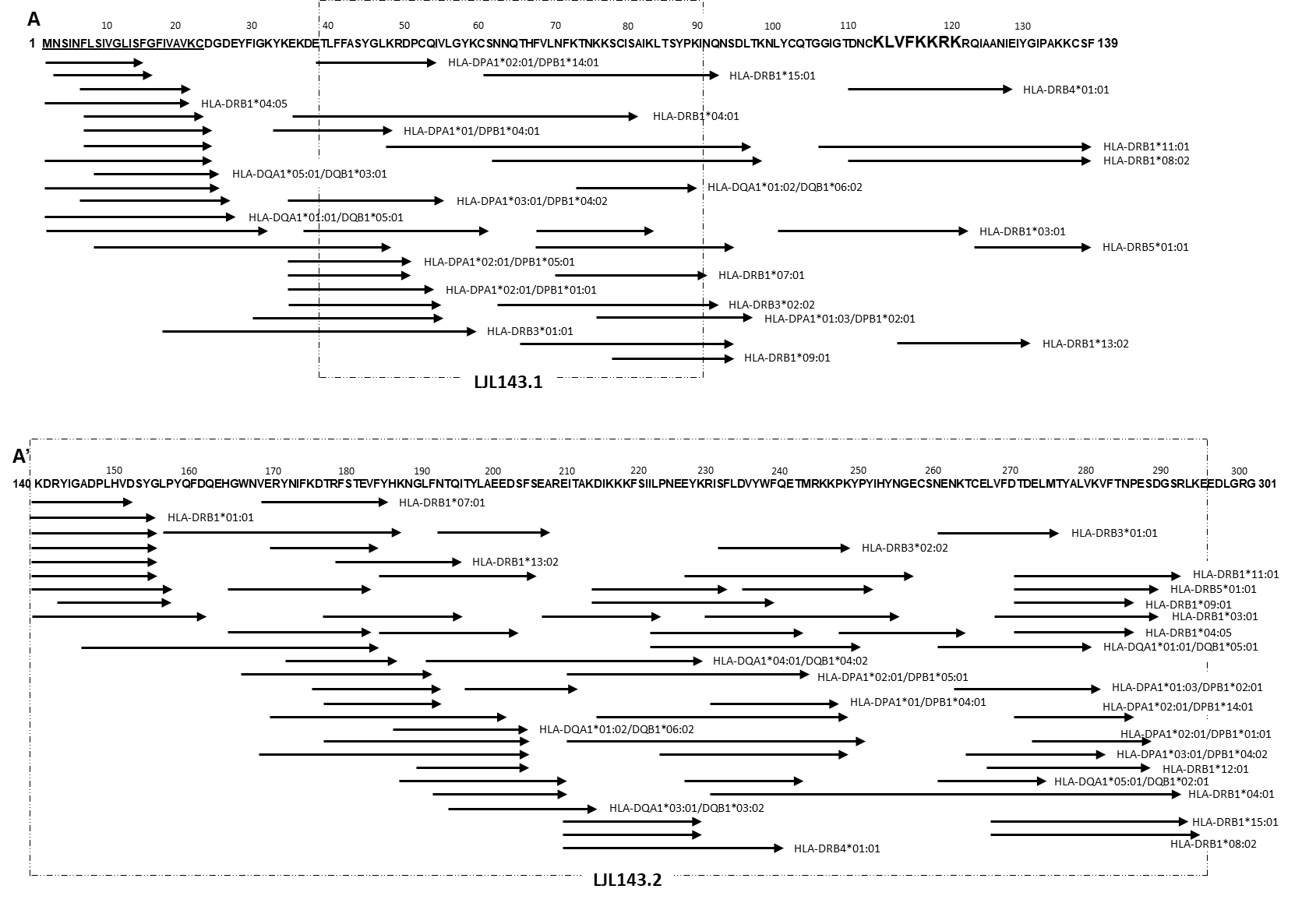


**Supplementary Figure 4. LJL-143 CD4+ T cell epitope mapping: extended representation of human MHC-II restricted epitopes.** LJL-143 (GenBank acc. no. AAS05319) CD4+ T cell epitope mapping was performed using the IEDB Analysis Resource considering 27 human MHC-II alleles. A/A’ represent the extended translation of the data set obtained (Supplementary data 12) and show predicted epitopes with percentile rank values between 2.5 and 7.5. Results are presented by allele. Each arrow represents one or more (contiguous) predicted epitopes. Underlined protein residues represent the signal peptide sequence. Magnified protein residues are potentially important for protein biological activity. Dashed boxes represent the two protein portions selected to be part of the final chimeric sand fly salivary antigen.

**
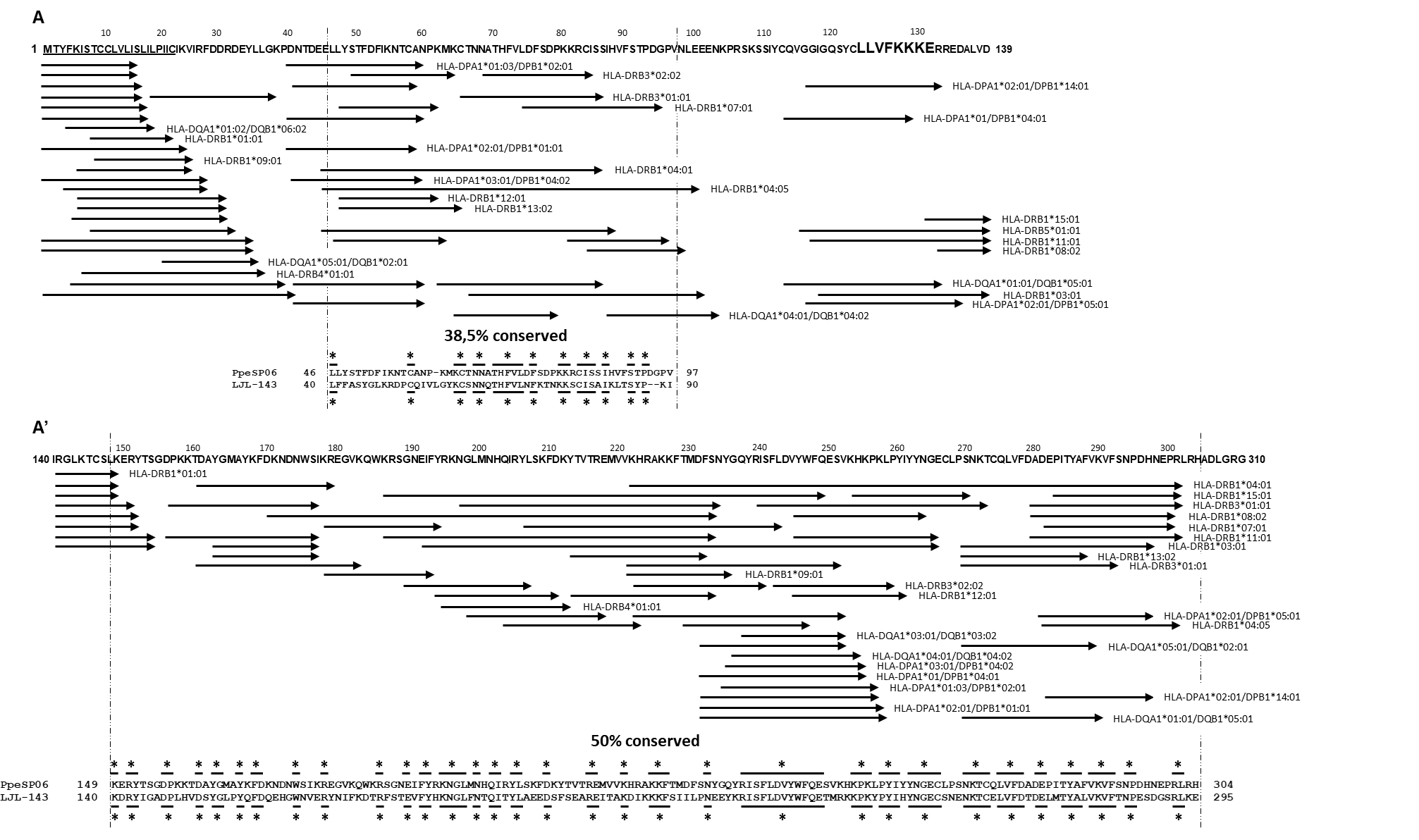
**

**Supplementary Figure 5. *in silico* determination of the human MHC-II restricted epitopes of PpeSP06, a LJL-143 homologue present in the saliva of the most relevant vector of Visceral Leishmaniasis in the Mediterranean Basin.** PpeSP06 (GenBank acc. no. DQ153100) CD4+ T cell epitope mapping was performed using the IEDB Analysis Resource considering 27 human MHC-II alleles. A/A’ represent the translation of the data set obtained (Supplementary data 13) and show the top 7.5% hits. Results are presented by allele. Each arrow represents one or more (contiguous) predicted epitopes. Underlined protein residues represent the signal peptide sequence. Magnified protein residues are potentially important for protein biological activity. Dashed vertical lines limit the homologous regions to LJL-143 portions selected to be part of the final chimeric sand fly salivary antigen. The alignment of PpeSP06 and LJL-143 portions of interest is represented, with the conserved residues highlighted. Percentage of amino acid sequence conservation within each portion was calculated and is also shown.

**
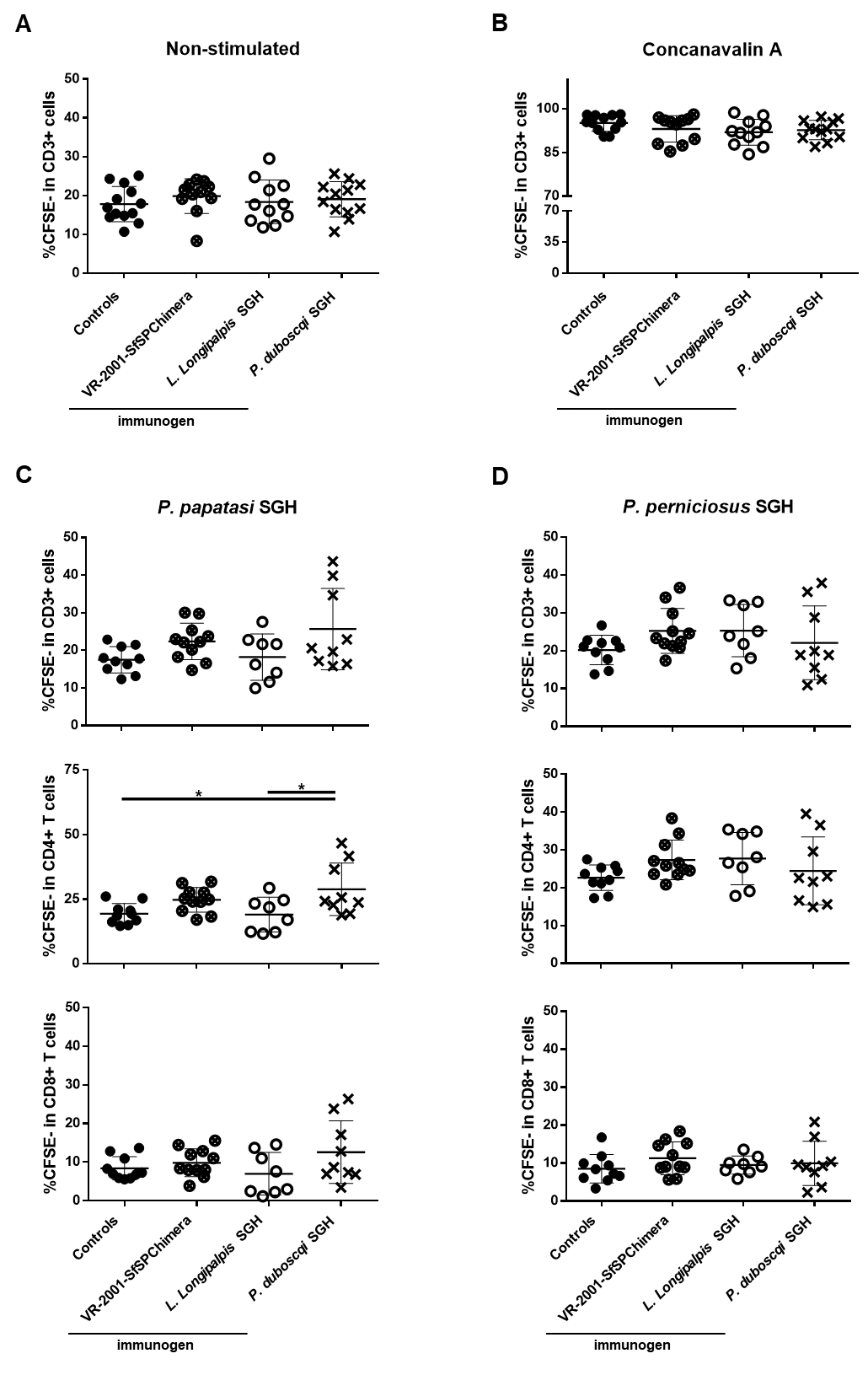
**

**Supplementary Figure 6. *Ex-vivo* cell proliferation experiments: controls and evaluation of the sand fly-derived DNA chimeric vaccine cross-reactive potential.** BALB/C mice were immunized intradermally in the right ear three times at two weeks intervals with 5 µg of VR-2001-SfSPChimera plasmid, or with either L. longipalpis or P. duboscqi SGH - the equivalent of 1 sand fly salivary gland pairs. Control animals received the same volume of the vehicle solution (PBS). One month after the last immunization, animals were euthanized, their spleens collected and processed to obtain CFSE-stained splenocytes suspensions. Frequencies of proliferating splenic T cells (total CD3+ and CD3+/CD4+ or CD3+/CD8+) were determined by flow cytometry after four days of culture in the presence of BMDCs (5:1 ratio), non-pulsed in the absence (A) or presence of 3 μg/ml concanavalin A (B), or pulsed with P. papatasi (C) or P. perniciosus (D) SGH (final concentration of 3 sand fly salivary gland pair/ml). Results from three independent experiments are shown. Each dot represents one animal. Average and SD of the values within each group are shown. Statistical differences are properly identified (One-Way ANOVA with Tukey´s *post hoc* analysis: * p≤0.05).
